# Supplementary material for: Large language models are proficient in solving and creating emotional intelligence tests
Source: Commun Psychol. 2025 May 21;3:80. doi: 10.1038/s44271-025-00258-x (PMC12095572; doi:10.1038/s44271-025-00258-x)
Supplement: Supplementary file 2 — supplementary material [file 44271_2025_258_MOESM2_ESM.pdf]

Supplementary Material for:

Large language models are proficient in solving and creating emotional intelligence tests

Supplementary Notes 1: ChatGPT conversations for generating new test items 2

STEM-B prompts & answers 2

STEU-B prompts & answers 4

GEMOK prompts & answers 9

GECo Emotion Regulation prompts & answers 15

GECo Emotion Management prompts & answers 19

Supplementary Notes 2: Final ChatGPT-created items 26

Final ChatGPT-generated items for STEM-B 26

Final ChatGPT-generated items for STEU-B 27

Final ChatGPT-generated items for GEMOK Blends 28

Final ChatGPT-generated items for GECo Emotion Regulation 32

Final ChatGPT-generated items for GECo Emotion Management 34

Supplementary Notes 3: Similarity rating study full results 39

Supplementary Table 1 40

Supplementary Table 2 41

Supplementary Table 3 42

Supplementary Table 4 43

Supplementary Table 5 44

Supplementary Table 6 45

Supplementary Table 7 46

Supplementary Table 8 47

Supplementary Notes 4: Item-level characteristics for ChatGPT-generated and original tests 48

Supplementary Table 9a 49

Supplementary Table 9b 50

Supplementary Table 10a 51

Supplementary Table 10b 52

Supplementary Table 11a 53

Supplementary Table 11b 54

Supplementary Table 12a 55

Supplementary Table 12b 56

Supplementary Table 13a 57

Supplementary Table 13b 58

Supplementary Notes 5: Similarities and differences between LLMs and human test-takers in solving ability EI items 59

Supplementary Notes 1: ChatGPT conversations for generating new test items

STEM-B prompts & answers

[First, ChatGPT 4 was presented with the original test items and given the following instructions:

Hi ChatGPT! Please solve all items of the following test:

Instructions: In this test, you will be presented with a few brief details about an emotional situation, and asked to choose from four responses the most effective course of action to manage both the emotions the person is feeling and the problems they face in that situation. Although more than one course of action might be acceptable, you are asked to choose what you think the most effective response for that person in that situation would be. Remember, you are not necessarily choosing what you would do, or the nicest thing to do, but choosing the most effective response for that situation.

Test items: 44 items]

PROMPT: Please generate 18 analogous items with the respective response options and solutions. Make sure that the items are equally distributed between the four emotions sadness, anger, fear, or disgust and the two domains workplace and personal life. Please also vary the effective strategies.

Sure, here are 18 analogous items distributed among the emotions of sadness, anger, fear, and disgust, and the domains of workplace and personal-life. The solutions take into account a variety of strategies:

1. **Emotion: Sadness, Domain: Workplace** Karen's favorite coworker, Sam, has been transferred to another branch, leaving Karen feeling quite lonely. What action would be the most effective for Karen? a) Get upset about Sam's transfer. b) Spend time with other coworkers and build new friendships. (Correct) c) Start looking for a new job. d) Hope that Sam will come back.
2. **Emotion: Anger, Domain: Personal-Life** Ted finds out that his best friend has been spreading rumors about him. What action would be the most effective for Ted? a) Confront his friend and demand an apology. (Correct) b) Cut off all communication with his friend. c) Start spreading rumors about his friend. d) Ignore it and continue as if nothing happened.
3. **Emotion: Fear, Domain: Workplace** Laura is nervous about an upcoming presentation she has to give in front of the company board. What action would be the most effective for Laura? a) Decide not to give the presentation. b) Find someone else to give the presentation. c) Practice the presentation and seek feedback to improve. (Correct) d) Just wing it and hope for the best.
4. **Emotion: Disgust, Domain: Personal-Life** Dave's neighbor's dog keeps leaving messes in his yard. What action would be the most effective for Dave? a) Return the mess to his neighbor's yard. b) Confront his neighbor and express his concern about the issue. (Correct) c) Ignore it and clean up the mess himself. d) Report the neighbor to the police.
5. **Emotion: Sadness, Domain: Personal-Life** Sophia's long-term relationship just ended. What action would be the most effective for Sophia? a) Dwell on the break-up and cry it out. b) Reach out to friends

and family for support. (Correct) c) Start dating immediately. d) Ignore her feelings and pretend everything is okay.

6. **Emotion: Anger, Domain: Workplace** Michael's idea was stolen by a colleague, who then received recognition for it. What action would be the most effective for Michael? a) Start a fight with the colleague. b) Talk to his superior about the situation. (Correct) c) Resent his colleague silently. d) Steal an idea from the colleague in return.
7. **Emotion: Fear, Domain: Personal-Life** Lily has been experiencing health problems and is afraid of the doctor's diagnosis. What action would be the most effective for Lily? a) Avoid going to the doctor. b) Try to self-diagnose using the internet. c) Go to the doctor to understand what's happening and discuss her fears. (Correct) d) Ignore her symptoms and hope they will go away.
8. **Emotion: Sadness, Domain: Workplace** Lucy has been passed over for a promotion she was expecting. What action would be the most effective for Lucy? a) Resent her boss for not promoting her. b) Quit her job. c) Seek feedback and work on her professional growth. (Correct) d) Avoid work and give minimal effort.
9. **Emotion: Anger, Domain: Personal-Life** Neil discovered that his brother used his car without asking and damaged it. What action would be the most effective for Neil? a) Refuse to talk to his brother. b) Seek reparations from his brother and discuss the importance of respect for personal property. (Correct) c) Damage his brother's property in retaliation. d) Ignore the damage and let it go.
10. **Emotion: Fear, Domain: Workplace** Olivia fears public speaking and is asked to lead a team meeting. What action would be the most effective for Olivia? a) Refuse to lead the meeting. b) Prepare thoroughly and practice her presentation. (Correct) c) Ask someone else to speak on her behalf. d) Wing it on the day of the meeting.
11. **Emotion: Disgust, Domain: Personal-Life** Max's roommate consistently leaves dirty dishes around the house. What action would be the most effective for Max? a) Start throwing out the dishes. b) Confront his roommate about the issue and suggest a cleaning schedule. (Correct) c) Move out immediately. d) Clean the dishes himself every time.
12. **Emotion: Sadness, Domain: Personal-Life** Julia's child has just moved away for college, leaving her feeling lonely. What action would be the most effective for Julia? a) Try to persuade her child to stay home. b) Find activities she enjoys to fill her time and stay connected with her child. (Correct) c) Sit at home and mourn her loneliness. d) Decide to have another child to replace the one who left.
13. **Emotion: Anger, Domain: Workplace** Ben's supervisor takes credit for his work during a meeting. What action would be the most effective for Ben? a) Publicly accuse his supervisor of stealing his work. b) Discuss the issue privately with his supervisor or report it to higher management. (Correct) c) Do poor work in future projects. d) Quit his job without notice.
14. **Emotion: Fear, Domain: Personal-Life** Susan is anxious about flying and has a trip coming up. What action would be the most effective for Susan? a) Cancel the trip and stay home. b) Learn about the safety of air travel and consider relaxation techniques. (Correct) c) Drink alcohol to calm her nerves. d) Just ignore her fear and hope it will go away.
15. **Emotion: Disgust, Domain: Workplace** Tony is assigned to a project he finds morally objectionable. What action would be the most effective for Tony? a) Do the project anyway. b) Complain about the project to his coworkers. c) Discuss his concerns with his supervisor or consider if the job aligns with his values. (Correct) d) Sabotage the project.
16. **Emotion: Sadness, Domain: Workplace** Anna's team didn't win the company's project competition, which she was very invested in. What action would be the most effective for Anna? a) Blame her team members for the loss. b) Use the experience as a learning opportunity to improve for next time. (Correct) c) Give up participating in future competitions. d) Demand a rematch.

17. **Emotion: Anger, Domain: Personal-Life** Eric found out his spouse forgot their anniversary. What action would be the most effective for Eric? a) Ignore his spouse until they realize what they forgot. b) Express his feelings about the forgotten anniversary and discuss ways to avoid such oversights. (Correct) c) Forget his spouse's birthday in return. d) Divorce his spouse over the oversight.
18. **Emotion: Fear, Domain: Workplace** Rachel is afraid of conflict and her colleagues are having a heated argument. What action would be the most effective for Rachel? a) Avoid the colleagues who are arguing. b) Take a stand and share her viewpoint in a constructive manner. (Correct) c) Take sides and escalate the argument. d) Start a rumor about the colleagues to distract them.

STEU-B prompts & answers

[First, ChatGPT 4 was presented with the original test items and given the following instructions:

“Hi ChatGPT! Please solve all items of the following test: Instructions

The following questions each describe a situation and ask you to choose which of five emotions is most likely to result from that situation. Here is an example:

Clara receives a gift. Clara is most likely to feel?

(a) happy (b) angry (c) frightened (d) bored (e) hungry

If you think Clara would feel happy, you would mark option A and then move to the next question. There are 42 questions.”]

PROMPT: Please generate 19 analogous items with the respective solutions. Use the emotions from the original test and create fictitious protagonists. Create 15 items with emotions as response options, and 4 with reversed format, such as in items 10, 19, or 20. Make the items challenging by adding one false response option that is close to the correct response, with a total of 5 response options.

RESPONSE: Alright, here are 19 analogous items with their respective solutions. For items with reversed format, the correct answers will be situations where the given emotion is a probable response.

Items with Emotions as Response Options

Item 43.

Nina watched as her younger brother won a trophy for his achievements in science. a) Anger b) Pride ✓ c) Fear d) Disgust e) Sadness

Item 44.

Marco's face turned red as his peers laughed at his mistaken answer. a) Joy b) Relief c) Anger d) Distress e) Embarrassment ✓

Item 45.

Sharon felt an overwhelming sense of warmth as she watched the stranger return her lost wallet. a) Gratitude ✓ b) Anger c) Distress d) Contempt e) Pride

Item 46.

Raul felt a rush of cold air and his heartbeat quicken as he heard footsteps behind him in the dark alley. a) Joy b) Fear ✓ c) Gratitude d) Pride e) Relief

Item 47.

Tiffany glared at her neighbor with a feeling of deep resentment as he played loud music late into the night. a) Joy b) Gratitude c) Anger ✓ d) Hope e) Relief

Item 48.

Chris felt a wave of contentment as he gazed at the painting he had spent weeks creating. a) Pride ✓ b) Anger c) Disgust d) Fear e) Sadness

Item 49.

Sophia's chest tightened and her eyes welled up as she read the final lines of the heartbreaking novel. a) Joy b) Sadness ✓ c) Relief d) Disgust e) Hope

Item 50.

As Leo saw the homeless puppy in the rain, he felt a profound desire to protect and shelter it. a) Disgust b) Gratitude c) Compassion ✓ d) Contempt e) Anger

Item 51.

Liam's heart sank as he read the rejection letter from his dream university. a) Joy b) Relief c) Pride d) Disgust e) Disappointment ✓

Item 52.

Jenny felt elated and couldn't stop smiling as she crossed the finish line. a) Sadness b) Anger c) Joy ✓ d) Fear e) Distress

Item 53.

Tom felt waves of annoyance when he found his younger brother had borrowed his laptop without permission again. a) Relief b) Joy c) Irritation ✓ d) Gratitude e) Hope

Item 54.

Victoria felt a sudden release of tension when she heard her family was safe after the hurricane. a) Fear b) Anger c) Relief ✓ d) Disgust e) Sadness

Item 55.

John felt a surge of hopefulness when he saw the first sprout in his garden after weeks of waiting. a) Disgust b) Anger c) Fear d) Hope ✓ e) Sadness

Item 56.

Olivia felt a sense of contentment and fulfillment as she looked at the happy faces of the children she volunteered to help. a) Anger b) Disgust c) Sadness d) Gratitude e) Satisfaction ✓

Item 57.

Ryan felt a pang of shame and remorse after realizing the hurtful impact of his words on his friend. a) Pride b) Guilt ✓ c) Joy d) Hope e) Gratitude

---

Items with Reversed Format (Situations as Response Options)

Item 58.

Which situation is most likely to make one feel contempt? a) Being awarded a medal of honor b) Witnessing someone cheat in a game ✓ c) Receiving a surprise gift d) Finding a lost item e) Achieving a long-term goal

Item 59.

In which situation is someone most likely to feel joy? a) Failing an important exam b) Getting caught in a heavy rain without an umbrella c) Losing a wallet with valuable items d) Witnessing an act of cruelty e) Reuniting with a long-lost friend ✓

Item 60.

In which situation is someone most likely to feel relieved? a) Learning a loved one is in the hospital b) Discovering a dent on a brand new car c) Missing a flight for a crucial meeting d) Finding out that a missing pet has been found safe ✓ e) Being informed of a sudden job loss

Item 61.

In which situation is someone most likely to feel grateful? a) Being publicly humiliated b) Having a meal ruined by poor service c) Being helped by a stranger during a medical emergency ✓ d) Learning about a betrayal by a close friend e) Getting stranded due to a vehicle breakdown

PROMPT: Thanks! These items are too easy, because in most cases there is only one viable response option. Please generate new response options for each item, where one or two wrong responses are similar to the correct response, but still wrong. For the first 15 items, only use words from the following list as response options: 1. Anger / Angry 2. Anxiety 3. Ashamed 4. Confused 5. Contempt / Contemptuous 6. Depressed 7. Dislike 8. Distress / Distressed 9. Excitement 10. Fear 11. Frustrated 12. Gratitude 13. Guilty 14. Hope 15. Horror 16. Jealousy 17. Joy 18. Pride 19. Rage 20. Regret 21. Relief / Relieved 22. Sad / Sadness 23. Scared 24. Shame 25. Surprise / Surprised Do not use any of these emotion words in the scenarios. If necessary, exchange single words in the scenarios so that these emotion words do not appear there.

RESPONSE: Certainly, I understand you're looking for more challenging items with response options that are closely related to the context. Here are revised items based on your specifications:

Items with Emotions as Response Options

Item 43.

Nina watched as her younger sibling received a trophy for remarkable achievements in science. a) Jealousy b) Pride ✓ c) Contempt d) Excitement e) Surprise

Item 44.

Marco's face turned red as his classmates chuckled at his incorrect response. a) Shame b) Anger c) Confusion d) Distress ✓ e) Frustration

Item 45.

Sharon felt an overwhelming sense of warmth as she watched the stranger return her misplaced wallet. a) Relief b) Gratitude ✓ c) Surprise d) Confusion e) Excitement

Item 46.

Raul felt a rush of cold air and his heartbeat quicken as he heard footsteps behind him in the dimly lit alley. a) Anxiety b) Fear ✓ c) Surprise d) Distress e) Horror

Item 47.

Tiffany glared at her neighbor with deep resentment as he played loud music until the early hours. a) Anger ✓ b) Frustration c) Distress d) Contempt e) Jealousy

Item 48.

Chris felt a wave of contentment as he gazed at the artwork he had spent weeks perfecting. a) Pride ✓ b) Joy c) Relief d) Surprise e) Gratitude

Item 49.

Sophia's chest tightened and her eyes welled up as she reached the concluding passages of the poignant novel. a) Sadness ✓ b) Distress c) Regret d) Confusion e) Surprise

Item 50.

As Leo noticed the abandoned puppy in the rain, he felt a profound desire to provide it with shelter. a) Sadness b) Compassion c) Relief d) Distress ✓ e) Regret

Item 51.

Liam's spirits plummeted as he read the rejection notice from his dream university. a) Sadness b) Regret c) Frustration d) Disappointment ✓ e) Shame

Item 52.

Jenny couldn't keep the smile off her face as she crossed the finish line, feeling elated. a) Excitement b) Joy ✓ c) Pride d) Relief e) Surprise

Item 53.

Tom felt waves of annoyance when he discovered his younger brother had again borrowed his laptop without asking. a) Frustration ✓ b) Anger c) Jealousy d) Contempt e) Distress

Item 54.

Victoria felt a sudden release of tension when she was informed her family was unharmed following the hurricane. a) Relief ✔ b) Gratitude c) Surprise d) Hope e) Excitement

Item 55.

John's spirits lifted when he noticed the first sprout in his garden after weeks of anticipation. a) Surprise b) Hope ✔ c) Excitement d) Joy e) Gratitude

Item 56.

Olivia experienced a sense of fulfillment seeing the bright smiles of the children she had volunteered to assist. a) Gratitude b) Pride c) Joy d) Relief e) Satisfaction ✔

Item 57.

Ryan experienced a pang of discomfort and self-reproach after realizing the hurtful consequences of his remarks on his friend. a) Guilt ✔ b) Shame c) Regret d) Sadness e) Anger

PROMPT: Thanks! Now, revise items 58-61 by making them more similar in style to the following original items from the test. As the target emotion in the scenario, use you can use items from the following list: 1. Anger / Angry 2. Anxiety 3. Ashamed 4. Confused 5. Contempt / Contemptuous 6. Depressed 7. Dislike 8. Distress / Distressed 9. Excitement 10. Fear 11. Frustrated 12. Gratitude 13. Guilty 14. Hope 15. Horror 16. Jealousy 17. Joy 18. Pride 19. Rage 20. Regret 21. Relief / Relieved 22. Sad / Sadness 23. Scared 24. Shame 25. Surprise / Surprised

Provide the correct answer for each item. Provide options that could plausibly evoke a feeling that is similar to the target emotion, but with one correct option that aligns most directly with the emotion in question.

Example items: 19. Megan is looking to buy a house. Something happened and she felt regret. What is most likely to have happened? (a) She didn't make an offer on a house she wanted, and now she is trying to find out if it is too late. (b) She found a house she liked that she didn't think she would find. (c) She couldn't make an offer on a house she liked because the bank didn't get her the money in time. (d) She didn't make an offer on a house she liked and now someone else has bought it. (e) She made an offer on a house and is waiting to see if it is accepted. 20. Mary was working at her desk. Something happened that caused her to feel surprised. What is most likely to have happened? (a) Her work-mate told a silly joke. (b) She was working on a new task she hadn't dealt with before. (c) She found some results that were different from what she thought they would be. (d) She realized she would not be able to complete her work. (e) She had to do a task she didn't normally do at work. 29. Quan and his wife are talking about what happened to them that day. Something happened that caused Quan to feel surprised. What is most likely to have happened? (a) His wife talked a lot, which did not usually happen. (b) His wife talked about things that were different to what they usually discussed. (c) His wife told him that she might have some bad news. (d) His wife told Quan some news that was not what he thought it would be. (e) His wife told a funny story. 41. Matthew has been at his current job for six months. Something happened that caused him to feel regret. What is most likely to have happened? (a) He did not apply for a position he wanted, and has found out that someone else less qualified got the job. (b) He did not apply for a position he wanted, and has started looking for a similar position. (c) He found out that opportunities for promotion have dried up. (d) He found out that he didn't get a position he thought he would get. (e) He didn't hear about a position he could have applied for and now it is too late. 10. Somebody is most likely to feel surprised after? (a) Something unexpected happens. (b) Something unfamiliar happens. (c) Something unusual happens. (d) Something scary happens. (e) Something silly happens.

Response:

Item 58.

Alex was walking home from work. Something happened that caused him to feel fear. What is most likely to have happened? (a) He remembered he had left his front door unlocked. (b) A group of people were laughing loudly in the distance. (c) A stray dog barked at him but was held back by a fence. (d) He heard footsteps closely following him in a dark alley. ✔ (e) He found out that he left his office keys at his desk.

Item 59.

Lucia was browsing her emails. Something happened that made her feel excitement. What is most likely to have happened? (a) She saw an email reminding her of a meeting tomorrow. (b) She received an invitation to a weekend getaway with her best friends. ✔ (c) She found an old email from a coworker sharing a recipe. (d) She received a newsletter about a workshop she might attend. (e) She got an email notifying her of a software update.

Item 60.

Carlos was at a community gathering. Something occurred that made him feel contempt. What is most likely to have happened? (a) He heard someone praising the event organizer. (b) He learned about the charitable activities of the community. (c) Someone spilled a drink on him by accident. (d) He overheard someone rudely and baselessly criticizing his close friend. ✔ (e) He got introduced to a new neighbor.

Item 61.

Sarah was reading a letter from her old school. Something in it made her feel ashamed. What is most likely to have happened? (a) The school was inviting alumni for a reunion. (b) The letter described the achievements of her batchmates. (c) She was reminded of an outstanding fee she never paid. ✔ (d) The school asked for donations for a new building. (e) The school was closing down due to financial difficulties.

I have made the scenarios more analogous to the style of the example items and incorporated emotions from the provided list, ensuring that each item's correct answer aligns most directly with the emotion in question.

GEMOK prompts & answers

[First, ChatGPT 4 was presented with the original test items and given the following instructions:

Hi ChatGPT! Please solve all items of the following test:

Instruction: In the following questionnaire you will be asked to read scenarios in which a fictional person is experiencing an emotional state that involves two different feelings. These feelings can occur together at the same time or consecutively one after the other. After each scenario, you will see five pairs of emotion words. Your task is to choose which of the emotion pairs best describes the feelings of the person in each scenario. There are 20 scenarios in total.]

PROMPT: Please generate 20 analogous scenarios with the respective response options and solutions. Make sure that the response options consist of a combination of 2 emotions from the following list: Pride, joy, happiness, pleasure, interest, anxiety, sadness, irritation, fear, disgust, anger, guilt, shame, contempt, jealousy, and surprise. Do not include the emotion words from the solution in the scenario. Make the scenarios between 5 and 7 sentences long.

Anna attends her school reunion. At the event, she overhears some old classmates talking about her recent success as an author. They don't realize she's listening. One mentions how they never expected Anna to do well, while another admits they're impressed with her achievements. Anna feels a rush of emotions, both wanting to confront them and yet feeling validated about her career.

- Pride and irritation
- Joy and anger
- Surprise and happiness
- Interest and contempt
- Sadness and pleasure

  Solution: Pride and irritation

Ben watches his son's piano recital. His son stumbles on some notes, but then manages to finish strong. Ben remembers his own failures as a musician and feels mixed emotions as the audience applauds his son's effort.

- Sadness and pride
- Anger and interest
- Joy and disgust
- Fear and pleasure
- Anxiety and happiness

  Solution: Sadness and pride

Clara discovers a secret letter from her partner to another person. As she reads through, she realizes that it's about a surprise birthday party for her. The planning details reflect the enormous effort her partner has put in.

- Joy and jealousy
- Surprise and guilt
- Interest and anxiety
- Happiness and anger
- Pleasure and irritation

  Solution: Surprise and guilt

David is a teacher, reviewing exam papers. He comes across an exceptionally well-written essay but then notices familiar phrases from a known publication. Realizing one of his top students has plagiarized, he's conflicted.

- Interest and anger
- Joy and disgust
- Shame and happiness
- Sadness and pride
- Contempt and anxiety

  Solution: Contempt and anxiety

Emma sees her childhood friend on TV, now a famous actress. She remembers their shared dreams of fame, but while Emma chose a different path, her friend pursued acting. As Emma sees her friend thank her parents in a speech, mixed feelings rise.

- Jealousy and joy
- Sadness and pride
- Interest and anger
- Happiness and irritation
- Fear and pleasure

  Solution: Jealousy and joy

Frank attends a friend's art exhibition. He notices one painting strikingly similar to a piece he made years ago. The painting receives numerous compliments, leaving Frank torn.

- Pride and disgust
- Interest and anxiety
- Sadness and joy
- Anger and pleasure
- Contempt and happiness

  Solution: Contempt and happiness

Grace finds an old journal of her mother's. Reading through, she discovers struggles and challenges her mother faced but never spoke about. Grace gains a deeper understanding and appreciation of her mother's life.

- Sadness and interest
- Joy and surprise
- Pride and anxiety
- Happiness and fear
- Anger and pleasure

Solution: Sadness and interest

Henry receives an email about a postponed project approval. This delay could hamper his promotion. Later, he finds out it's due to a senior colleague wanting to include Henry's ideas for a broader audience.

- Surprise and irritation
- Interest and anger
- Joy and jealousy
- Anxiety and pride
- Fear and happiness

Solution: Anxiety and pride

Isabelle sees her brother's name in a scandalous news article. Upon reading, she learns it's a positive piece on him stopping a bad event from happening, but the headline was misleading.

- Anger and pride
- Joy and disgust
- Fear and interest
- Surprise and sadness
- Pleasure and anxiety

Solution: Anger and pride

Jack listens to his daughter practicing her speech. She makes multiple mistakes, but her dedication reminds him of his childhood determination.

- Sadness and joy
- Pride and fear
- Surprise and irritation
- Disgust and happiness
- Interest and anger

Solution: Sadness and joy

Karen receives a gift from an anonymous sender. Unwrapping it, she finds a cherished book from her childhood. Memories flood back, both pleasant and painful.

- Surprise and sadness
- Joy and fear
- Pride and anxiety
- Happiness and contempt
- Disgust and interest

Solution: Surprise and sadness

Liam watches a documentary on a global issue. He's deeply affected by the real-life stories and feels the urge to contribute to the cause.

- Sadness and interest
- Anger and pleasure
- Joy and anxiety
- Fear and pride
- Disgust and happiness

Solution: Sadness and interest

Monica attends her best friend's wedding. Seeing her friend in a wedding dress, she recalls the promises they made to each other as kids.

- Joy and jealousy
- Sadness and pride
- Happiness and fear
- Interest and disgust
- Surprise and contempt

Solution: Joy and jealousy

Nathan finds a lost puppy near his house. He puts up posters and when the owner arrives, Nathan realizes it's someone who once wronged him.

- Disgust and interest
- Anger and pleasure
- Fear and joy
- Shame and happiness
- Sadness and pride

Solution: Disgust and interest

Olivia gets feedback from her boss about her project. She's praised for her efforts but is also made aware of crucial errors she overlooked.

- Pride and anxiety
- Joy and fear
- Anger and happiness
- Surprise and sadness
- Disgust and interest

Solution: Pride and anxiety

Peter receives an invitation to an exclusive event. As he's preparing, he learns it was sent by mistake and was meant for someone else.

- Surprise and sadness
- Anger and pride
- Interest and disgust
- Fear and joy
- Shame and pleasure

Solution: Shame and pleasure

Quinn reads a social media post mocking her recent achievements. However, in the comments, several strangers defend her and praise her accomplishments.

- Anger and pride
- Joy and jealousy
- Surprise and fear
- Sadness and happiness
- Disgust and interest

Solution: Anger and pride

Rachel visits her grandparents and stumbles upon old photos. She sees her grandfather receiving an award she knew nothing about and feels a deeper connection.

- Interest and joy
- Sadness and pride
- Surprise and contempt
- Happiness and disgust
- Fear and pleasure

Solution: Interest and joy

Steve joins a fitness challenge at work. He pushes hard but finishes second. However, the winner admits they were inspired by Steve's dedication.

- Sadness and pride

- Disgust and happiness
- Surprise and anxiety
- Fear and interest
- Anger and joy

  Solution: Sadness and pride

Tara finds her child drawing on the walls. The drawings, though messy, showcase her child's immense creativity and imagination.

- Anger and pleasure
- Disgust and pride
- Fear and interest
- Surprise and sadness
- Joy and contempt

  Solution: Disgust and pride

PROMPT: Please make all 20 scenarios, but not the response options, a bit longer. Do not include the emotion words from the solution in the scenario.

Of course! Let's expand on the scenarios:

1. **Anna attends her school reunion.** The room buzzes with nostalgic laughter and chatter. As she moves to get a drink, she overhears some old classmates gathered in a corner. They discuss her recent success as an author. They don't realize she's nearby. One mentions how they never expected Anna to do so well, while another admits being impressed by her achievements.
2. **Ben watches his son's piano recital.** The auditorium is filled with expectant parents and well-wishers. His son begins with confidence but stumbles on some notes midway. Ben's heart skips a beat, memories of his own failures as a musician flooding back. However, his son regains composure, finishing with a flourish, earning applause from the audience.
3. **Clara discovers a secret letter tucked in a drawer.** The handwriting belongs to her partner. As she reads through, the content mentions another person. But, as she delves deeper, she realizes it's about organizing a surprise birthday party for her. The intricate planning details reflect the enormous effort her partner has invested.
4. **David, a dedicated teacher, sits in his study reviewing exam papers.** The room is silent except for the rustle of pages. An exceptionally well-written essay catches his attention. Yet, as he reads on, he identifies familiar phrases from a renowned publication. The realization that one of his brightest students might have plagiarized makes him pause.
5. **Emma casually flips through channels on a lazy afternoon.** She stops abruptly when she recognizes a familiar face. It's her childhood friend, now a famous actress, being interviewed. Emma recalls their shared dreams of fame, and while she opted for a different life, her friend chased the limelight. As her friend thanks her parents in a speech, Emma's past and present collide.
6. **Frank, an artist, attends a friend's art exhibition downtown.** The gallery is filled with art enthusiasts. While moving from one piece to another, Frank's eyes fall on a painting that seems strikingly similar to a creation he made years ago. As the evening progresses, the painting garners considerable attention and compliments, leaving Frank in introspection.
7. **Grace, while cleaning the attic, finds an old, dusty journal.** The cover indicates it belonged to her mother. Flipping through the pages, Grace discovers struggles and challenges her mother faced but never vocalized. Each entry gives Grace a deeper understanding of the sacrifices her mother made.

8. **Henry, immersed in work, receives an unexpected email notification.** The subject suggests a postponed project approval, which he had been eagerly awaiting. He realizes this delay could jeopardize his upcoming promotion. Yet, later in the day, he discovers the postponement was due to a senior colleague wanting to include Henry's innovative ideas for a broader implementation.

9. **Isabelle's morning coffee is interrupted by a sensational news headline on TV.** Her brother's name flashes in bold. A lump forms in her throat, but as she listens, she learns it's a positive story about him preventing a scandalous event, despite the misleading headline.

10. **Jack sits on his porch, sipping tea as he listens to his daughter practicing her speech indoors.** The pauses, retries, and stumbles remind him of his own challenges growing up. Her relentless dedication, despite the mistakes, takes him on a trip down memory lane.

11. **Karen, arriving home after a tiring day, spots a package at her doorstep.** With no sender's name, she curiously unwraps it. Inside is a cherished book from her childhood, bringing back a flood of memories, some pleasant and others tinged with pain.

12. **Liam, on a quiet evening, decides to watch a documentary.** The gripping real-life stories about a pressing global issue deeply move him. He's affected not just by the narratives but also by the raw emotions displayed, making him contemplate his role in the larger scheme of things.

13. **Monica, donning a beautiful bridesmaid dress, attends her best friend's wedding.** As she watches her friend walk down the aisle, memories of their childhood games and pretend weddings flash before her eyes. Their innocent promises of the past juxtapose with the reality of the present.

14. **Nathan, out on his morning walk, discovers a lost puppy whimpering near his house.** He takes the initiative to find its owner, putting up posters and posts on social media. When the owner finally arrives, Nathan's taken aback — it's someone from his past who had once wronged him.

15. **Olivia, engrossed in her work, is startled by a ping on her computer.** It's feedback from her boss about the project she submitted last week. The initial praises make her heart swell, but as she reads further, she's made aware of some overlooked errors that might have serious implications.

16. **Peter, on a serene evening, receives an elegant invitation card.** The glossy card mentions an exclusive event in the city's most upscale venue. He's thrilled but while preparing, a call informs him the invitation was sent by mistake and was intended for someone else with a similar name.

17. **Quinn, while browsing her social media feed, stumbles upon a post discussing her recent achievements.** The post, dripping with sarcasm, mocks her efforts. However, scrolling through the comments, she discovers an army of strangers ardently defending and praising her accomplishments.

18. **Rachel, during a summer visit to her grandparents' old house, stumbles upon a stack of old photos.** One particular photo catches her eye — her grandfather, much younger, receiving a prestigious award. The image paints a picture of a side of him she never knew, making her want to delve deeper into his past.

19. **Steve, eager to improve his health, joins a month-long fitness challenge at work.** Days of rigorous training culminate in a finale event. Despite giving his best, he finishes second. But the winner, during the award ceremony, candidly admits being inspired by Steve's unwavering dedication throughout the challenge.

20. **Tara, after a long phone call, walks into her living room to find her child busy with crayons.** The walls, previously pristine, now sport vibrant, albeit messy, drawings. While the initial shock is overwhelming, she can't help but notice the vivid imagination her child has showcased in every scribble.

GECo Emotion Regulation prompts & answers

[First, ChatGPT 4 was presented with the original test items and given the following instructions:

"Hi ChatGPT! Read the following items and choose the 2 options out of 4 (a-d) that represent what you would think in the respective scenario. Make a list with the item numbers and the letters of the options you chose."

This was done for 8 items at a time due to the character limitation in ChatGPT4.]

PROMPT: The responses to these items consist of 4 maladaptive cognitive strategies (blaming oneself, blaming others, rumination, and catastrophizing) and 5 adaptive cognitive strategies (acceptance, putting into perspective, positive refocusing, refocusing on planning, and positive reappraisal). Please generate 28 analogous items, referring to the emotions anger/irritation, anxiety/ fear, and sadness/ despair in the workplace. For each item, there must be 2 response options that represent adaptive strategies (acceptance, putting into perspective, positive refocusing, refocusing on planning, and positive reappraisal) and 2 response options that represent maladaptive strategies (blaming oneself, blaming others, rumination, and catastrophizing). Make sure to evenly distribute the various strategies to the items.

RESPONSE: Certainly! Here are 28 analogous items addressing the emotions of anger/irritation, anxiety/fear, and sadness/despair in the workplace, each with two adaptive and two maladaptive cognitive strategies as response options:

1. **You discover your proposal was rejected in favor of a colleague's idea, causing irritation.** a. You consider the feedback for future improvements. (positive reappraisal) b. You ponder why management always favors others. (rumination) c. You decide to discuss with your colleague for a combined approach next time. (refocusing on planning) d. You blame yourself for not presenting it better. (blaming oneself)
2. **An unexpected project delay makes you anxious about meeting the deadline.** a. You strategize a new approach to make up for lost time. (refocusing on planning) b. You think it's always your luck that projects get delayed. (catastrophizing) c. You see it as an opportunity to refine the final output. (positive refocusing) d. You dwell on every small mistake that led to this. (rumination)
3. **You overhear colleagues discussing your recent mistake, leading to feelings of sadness.** a. You decide to address the error in the next team meeting. (acceptance) b. You think they always find a reason to talk behind your back. (blaming others) c. You take a moment to think of your past successes. (positive refocusing) d. You constantly replay their words in your mind. (rumination)
4. **Your request for leave gets declined, causing frustration.** a. You reevaluate the importance of taking those specific days off. (putting into perspective) b. You blame HR for always being so inflexible. (blaming others) c. You think of alternative dates to apply for leave. (refocusing on planning) d. You feel this always happens when you really need a break. (catastrophizing)

... and so on.

Would you like me to continue with the remaining items?

PROMPT: Repeat these items but make the scenarios a bit longer while keeping the response options unchanged. Then continue with the next items.

RESPONSE: Of course, let's delve deeper into the scenarios.

1. **After spending weeks preparing a detailed proposal, you're taken aback to learn that it was overshadowed by a simpler idea from a colleague. This sudden revelation fills you with irritation.** a. You consider the feedback for future improvements. (positive reappraisal) b. You ponder why management always favors others. (rumination) c. You decide to discuss with your colleague for a combined approach next time. (refocusing on planning) d. You blame yourself for not presenting it better. (blaming oneself)

2. **An unforeseen complication arises in your project. The initial timeline, which was already tight, now seems impossible. Waves of anxiety rush over you as the looming deadline approaches.** a. You strategize a new approach to make up for lost time. (refocusing on planning) b. You think it's always your luck that projects get delayed. (catastrophizing) c. You see it as an opportunity to refine the final output. (positive refocusing) d. You dwell on every small mistake that led to this. (rumination)
3. **While taking a break, you accidentally overhear a couple of colleagues in the next room discussing your recent error in detail. The weight of their words bears down on you, immersing you in sadness.** a. You decide to address the error in the next team meeting. (acceptance) b. You think they always find a reason to talk behind your back. (blaming others) c. You take a moment to think of your past successes. (positive refocusing) d. You constantly replay their words in your mind. (rumination)
4. **You had been planning a family vacation for months. When you finally muster the courage to apply for leave, it gets declined due to some organizational priorities. The frustration is palpable.** a. You reevaluate the importance of taking those specific days off. (putting into perspective) b. You blame HR for always being so inflexible. (blaming others) c. You think of alternative dates to apply for leave. (refocusing on planning) d. You feel this always happens when you really need a break. (catastrophizing)
5. **Every year, your team has a chance to pitch new initiatives. This year, you had a groundbreaking idea, but upon presenting, it's met with disinterest. Anger bubbles up as you consider the hours of research and preparation.** a. You decide to seek feedback and refine the idea for next time. (refocusing on planning) b. You assume the team never really values your suggestions. (blaming others) c. You take solace in the fact that not every idea will be a hit and remain optimistic. (positive refocusing) d. You replay the presentation, pinpointing moments of potential failure. (rumination)
6. **The department undergoes an audit. While you've been diligent, a minor oversight from months ago resurfaces, drawing unwanted attention. The scrutiny and accompanying anxiety are almost paralyzing.** a. You recognize the need for stringent checks in the future. (acceptance) b. You can't shake off the feeling that someone intentionally highlighted the oversight. (blaming others) c. You decide to develop a checklist to avoid such lapses in the future. (refocusing on planning) d. You're consumed by the thought of what other errors might come to light. (catastrophizing)

I can continue crafting more of these scenarios with your guidance. Would you like me to proceed?

PROMPT: Yes, please proceed and remember to evenly distribute the various strategies across the items. For each item, there must be 2 response options that represent adaptive strategies (acceptance, putting into perspective, positive refocusing, refocusing on planning, and positive reappraisal) and 2 response options that represent maladaptive strategies (blaming oneself, blaming others, rumination, and catastrophizing).

RESPONSE: Certainly, let's continue.

7. **You've been working tirelessly on a project for months. Just as it's about to launch, management pulls the plug, citing shifting priorities. The disappointment is crushing.** a. You reflect on the learnings you've gained from the project regardless of its outcome. (positive reappraisal) b. You blame the top management for not recognizing the project's value. (blaming others) c. You think about how to repurpose your work for future projects. (refocusing on planning) d. You keep questioning your worthiness in the organization. (blaming oneself)
8. **Every team member was assigned a challenging client. Yours seems particularly difficult, always raising last-minute issues. Their demands keep you on your toes, constantly inducing fear.** a. You analyze their feedback to see if there's a pattern to anticipate future concerns. (refocusing on planning) b. You feel like you're always the one stuck with the problematic clients. (catastrophizing) c. You acknowledge that challenging clients can offer valuable lessons. (acceptance) d. You can't help but think others secretly enjoy watching you struggle. (blaming others)
9. **You've recently been promoted. With new responsibilities, you find yourself struggling to keep up. Colleagues you once considered friends seem distant, making you feel isolated and sad.** a. You take a step back to consider that everyone goes through adjustment phases. (putting into perspective) b. You start doubting if the promotion was a setup for failure. (rumination) c. You decide to attend a training or workshop to hone your skills for the new role. (positive refocusing) d. You blame yourself for not living up to the new expectations. (blaming oneself)

10. **Despite all your efforts, your department's monthly performance has been lagging for the third consecutive month. The mounting pressure and inevitable confrontations fill you with anxiety.** a. You strategize with your team on ways to pivot and make improvements. (refocusing on planning) b. You find yourself replaying past decisions, wondering where you went wrong. (rumination) c. You focus on the small wins and celebrate them to build morale. (positive refocusing) d. You can't shake off the belief that someone in the team might be sabotaging efforts. (blaming others)
11. **You present a new, innovative approach to a recurring problem in a meeting. However, it's met with immediate resistance from a senior member, causing public embarrassment and anger.** a. You seek private feedback to understand their concerns better. (positive reappraisal) b. You dwell on how your ideas are never given a fair chance. (catastrophizing) c. You remind yourself of the importance of diverse perspectives in refining ideas. (putting into perspective) d. You resentfully blame the senior member for always being stuck in their ways. (blaming others)
12. **An error in your financial reports gets highlighted during a review. It's a minor mistake but has big implications. The weight of this mistake drags you into despair.** a. You acknowledge the mistake and focus on implementing preventive measures. (acceptance) b. You keep pondering how you could be so careless. (blaming oneself) c. You decide to seek mentorship or training in financial reporting. (positive refocusing) d. You feel like these mistakes define your entire career. (catastrophizing)
13. **You've been leading a team virtually due to the current work-from-home scenario. The lack of physical interaction has its challenges, and you fear miscommunication and its repercussions.** a. You think of organizing virtual team-building exercises to enhance cohesion. (refocusing on planning) b. You start to believe that remote work is inevitably doomed to fail. (catastrophizing) c. You remind yourself that every new mode of working has its learning curve. (positive reappraisal) d. You blame the organization for not providing adequate training for remote work. (blaming others)

I've aimed to evenly distribute the various strategies across these scenarios. Let me know if any further adjustments or continuations are needed!

PROMPT: Please create more items and remember to evenly distribute the various strategies across the items. For each item, there must be 2 response options that represent adaptive strategies (acceptance, putting into perspective, positive refocusing, refocusing on planning, and positive reappraisal) and 2 response options that represent maladaptive strategies (blaming oneself, blaming others, rumination, and catastrophizing). Make sure each item is about a specific situation.

RESPONSE: Of course, let's continue with more items.

14. **A client sends a harsh email criticizing your team's work. You can't help but feel angered and questioned about your team's competency.** a. You consider discussing the feedback with the client to ensure future alignment. (refocusing on planning) b. You feel like your team is always at the receiving end of undue criticism. (catastrophizing) c. You evaluate the feedback to extract constructive points. (positive reappraisal) d. You blame a specific team member for not catching the issue earlier. (blaming others)
15. **You missed an important deadline, leading to a delay in project delivery. The oversight fills you with anxiety, anticipating the client's reaction.** a. You accept the oversight and think of ways to prevent it in the future. (acceptance) b. You can't stop ruminating over the consequences of this delay. (rumination) c. You brainstorm with your team about damage control and possible solutions. (positive refocusing) d. You blame yourself endlessly for not being more attentive. (blaming oneself)
16. **During a video call, a colleague unexpectedly challenges your solution. Their unexpected opposition throws you into a state of irritation.** a. You appreciate diverse viewpoints and plan a follow-up discussion. (positive reappraisal) b. You think they are intentionally trying to undermine you. (blaming others) c. You re-evaluate your proposal considering their points. (refocusing on planning) d. You feel like maybe you're not cut out for these kinds of discussions. (blaming oneself)
17. **Despite your hard work, another colleague gets the credit for a project's success. The oversight leaves you feeling overlooked and filled with despair.** a. You decide to communicate more proactively about your contributions in the future. (refocusing on planning) b. You ruminate about how your efforts always go unnoticed. (rumination) c. You focus on the project's success rather than who got the credit. (positive refocusing) d. You feel the management always favors certain individuals. (blaming others)

18. **You receive feedback that your presentation style lacks energy and enthusiasm. This catches you off-guard and fills you with sadness.** a. You see it as an opportunity to improve and consider joining a public speaking course. (positive refocusing) b. You believe that no matter what you do, it will never be enough. (catastrophizing) c. You reflect on previous presentations to understand this feedback better. (positive reappraisal) d. You blame yourself for not being naturally charismatic. (blaming oneself)
19. **A project you were passionate about gets discontinued due to budget cuts. The decision is disheartening and stirs feelings of anger.** a. You remind yourself of the broader business context and its challenges. (putting into perspective) b. You can't stop thinking about the wasted time and effort. (rumination) c. You think of ways to adapt the work done into another valuable initiative. (refocusing on planning) d. You believe that management doesn't value your contributions. (blaming others)
20. **You're assigned a new team member who frequently disagrees with your approaches. The constant disagreements induce feelings of anxiety.** a. You recognize the value of diverse perspectives in achieving optimal solutions. (acceptance) b. You feel the new team member is intentionally trying to sabotage your efforts. (blaming others) c. You consider scheduling regular check-ins to align on project objectives. (refocusing on planning) d. You start doubting your capabilities as a leader. (blaming oneself)

I hope these additional items meet your requirements. Let me know if you need any further scenarios or adjustments!

PROMPT: Please create 8 more items with new situations and remember to evenly distribute the various strategies across the items. For each item, there have to be 2 response options that represent adaptive strategies (acceptance, putting into perspective, positive refocusing, refocusing on planning, and positive reappraisal) and 2 response options that represent maladaptive strategies (blaming oneself, blaming others, rumination, and catastrophizing). Make sure each item is about a specific situation and make each scenario complex.

Absolutely, let's create 8 more intricate items:

21. **While leading a workshop, technical glitches disrupt the flow, leaving participants visibly frustrated. You feel a rising tide of anxiety about the potential fallout.** a. You acknowledge the hiccups and quickly switch to an alternative discussion until the issues are resolved. (refocusing on planning) b. You believe you should've foreseen and prevented every technical problem. (blaming oneself) c. You consider this an opportunity to showcase adaptability under pressure. (positive reappraisal) d. You continuously replay the attendees' irritated faces in your mind. (rumination)
22. **During a company retreat, you overhear colleagues discussing your recent project in a critical tone. The covert criticism fills you with sadness and anger.** a. You decide to approach them later for direct feedback to understand their concerns. (positive refocusing) b. You're certain that they've always had a vendetta against you. (blaming others) c. You remind yourself that everyone is entitled to their opinions, and it's essential to separate work from personal feelings. (putting into perspective) d. You fear this might be the general opinion behind your back. (catastrophizing)
23. **A promotion you had been eyeing goes to a less experienced colleague. This unexpected decision instills feelings of despair and confusion.** a. You seek feedback on areas of improvement and focus on your professional growth. (refocusing on planning) b. You think of the countless evenings you spent working late and feel it's all been in vain. (rumination) c. You congratulate your colleague, genuinely hoping they excel in the role. (acceptance) d. You're sure there's office politics at play here. (blaming others)
24. **Your proposal for a new initiative gets declined without much explanation. The abrupt dismissal leaves you feeling anxious and uncertain about your standing.** a. You take a step back and reflect on potential areas in your proposal that could be strengthened. (positive reappraisal) b. You constantly worry about how this rejection will impact your reputation. (rumination) c. You consult mentors and peers to refine your idea for a future pitch. (refocusing on planning) d. You believe decision-makers intentionally sideline your suggestions. (blaming others)
25. **Despite your best efforts, a client decides to end their contract early. This unforeseen setback fills you with anger and desolation.** a. You conduct a thorough review to learn from this experience for future client relationships. (positive refocusing) b. You are convinced the client had unrealistic expectations from the get-go. (blaming others) c. You remind yourself of the volatile nature of the

business world and use this as a stepping stone. (putting into perspective) d. You feel that maybe you're not cut out for this role. (blaming oneself)

26. **During a team-building activity, your idea gets overlooked in favor of another, making you feel insignificant and undervalued.** a. You respect the group's decision and actively participate in executing the chosen idea. (acceptance) b. You assume your team doesn't value your input. (catastrophizing) c. You think of ways to communicate your ideas more compellingly in future discussions. (refocusing on planning) d. You can't stop thinking about how your idea might have been better. (rumination)
27. **While presenting a quarterly report, you mistakenly share outdated figures, leading to a wave of confusion. The blunder brings feelings of intense embarrassment and fear.** a. You immediately correct the oversight and apologize for the confusion, ensuring accuracy in the future. (positive refocusing) b. You feel that such mistakes might cost you your job. (catastrophizing) c. You consider this a learning curve and think of implementing a verification process. (positive reappraisal) d. You blame the data team for not updating the figures timely. (blaming others)
28. **After a year of hard work, the product you've been developing receives mixed reviews post-launch. The criticism makes you question the entire project's worth.** a. You delve into the feedback, identifying areas that can be enhanced in subsequent versions. (refocusing on planning) b. You regret the time spent on this project, feeling it was all a waste. (rumination) c. You remind yourself of the innovation the product brings and its potential with some tweaks. (positive reappraisal) d. You believe certain team members didn't put in their best, leading to this outcome. (blaming others)

I hope these scenarios and response options are helpful. Let me know if you need any further scenarios or adjustments!

GECo Emotion Management prompts & answers

[First, ChatGPT 4 was presented with the original test items and given the following instructions:

“Hi ChatGPT! Please solve all items of the following test:”

Items were presented in 3 parcels due to the character limitation in ChatGPT 4.]

PROMPT: The response options for all of these test items consist of the five strategies for managing emotional situations: compromise, accommodation, competing, collaboration, avoidance. Please generate 20 analogous items with the respective response options and solutions. generate 4 items per strategy and use the other strategies as distractors. Ensure that the following criteria are met: 1) each scenario describes a concrete situation (not a general issue) and contains another person who feels/ expresses an emotion (anger/irritation, inappropriate happiness/joy, anxiety/fear, sadness/despair) and 2) the respondent's position (e.g., supervisor, co-worker, client, team member etc.) in each scenario is clear.

RESPONSE: Certainly! Here are 20 test items, each catering to one of the five strategies for managing emotional situations:

1. COMPROMISE: **Scenario:** Your team member, John, seems anxious about a shared task. He believes his way is faster, but you think yours is more efficient. **Options**: a) Propose combining elements of both methods. b) Tell John you will do it your way since you're the senior. c) Let John decide entirely. d) Discuss the benefits of both methods in a team meeting. e) Avoid the conversation and just get the task done as quickly as possible. **Answer**: a) Propose combining elements of both methods.

2. COMPROMISE: **Scenario:** Your coworker, Lisa, expresses irritation that your meetings always clash with her other commitments. **Options**: a) Propose alternating meeting times each week. b) Tell Lisa she should prioritize your meetings. c) Always adjust to Lisa's schedule. d) Collaboratively set up a joint calendar to find mutual availability. e) Don’t discuss the clash and hope she attends. **Answer**: a) Propose alternating meeting times each week.

3. COMPROMISE: **Scenario:** A client, Mr. Smith, is unhappy with the final design, suggesting it doesn't capture his vision. **Options**: a) Propose a design that merges both visions. b) Insist on the original design. c) Accept Mr. Smith's design completely. d) Arrange a workshop with Mr. Smith to co-create the design. e) Avoid any discussion and send the invoice. **Answer**: a) Propose a design that merges both visions.

4. COMPROMISE: **Scenario:** During a project, your team member, Rachel, is elated her strategy is generating good results. However, you notice potential long-term issues. **Options**: a) Propose tweaks to her strategy to avoid future pitfalls. b) Demand a change in strategy immediately. c) Adopt her strategy without question. d) Set up a brainstorming session to evolve the strategy further. e) Let it slide and hope the issues never arise. **Answer**: a) Propose tweaks to her strategy to avoid future pitfalls.

5. ACCOMMODATION: **Scenario:** Your co-worker, Tom, is irritated because he prefers working in a quiet environment, but you often have video calls. **Options**: a) Demand he gets noise-cancelling headphones. b) Offer to take calls in a meeting room to not disturb him. c) Combine both work patterns. d) Propose a team discussion about workspace norms. e) Just continue as normal, hoping he'll adjust. **Answer**: b) Offer to take calls in a meeting room to not disturb him.

6. ACCOMMODATION: **Scenario:** A client, Mrs. Lopez, feels anxious about a risky but potentially rewarding strategy you proposed. **Options**: a) Tell her to trust you completely. b) Switch to a more conservative strategy for her comfort. c) Seek a middle ground. d) Offer a joint strategy session to address concerns. e) Avoid discussing the strategy further. **Answer**: b) Switch to a more conservative strategy for her comfort.

7. ACCOMMODATION: **Scenario:** Your supervisor is upset about a decision you made without consulting them. **Options**: a) Defend your decision strongly. b) Apologize and offer to revert the decision. c) Find a compromise between your actions and their preference. d) Propose a feedback session for better decision-making in the future. e) Avoid discussing it and proceed with other tasks. **Answer**: b) Apologize and offer to revert the decision.

8. ACCOMMODATION: **Scenario:** At a company celebration, your junior colleague is overly ecstatic and making others uncomfortable. **Options**: a) Tell her to calm down immediately. b) Gently pull her aside and ask if she's okay, letting her express herself. c) Propose a middle-ground behavior. d) Organize a team game to channel the excitement. e) Avoid interacting with her for the evening. **Answer**: b) Gently pull her aside and ask if she's okay, letting her express herself.

9. COMPETING: **Scenario:** A team member, David, challenges your expertise in a meeting, hinting that his approach is superior. **Options**: a) Acknowledge his points and agree. b) Assert your expertise and experience, clarifying why your approach is right. c) Propose a blend of both methods. d) Organize a separate brainstorming session. e) Ignore his challenge and move on. **Answer**: b) Assert your expertise and experience, clarifying why your approach is right.

10. COMPETING: **Scenario:** Your client questions the pricing, suggesting it's too high for the value provided. **Options**: a) Immediately offer a discount. b) Firmly explain the value and why the price is justified. c) Propose a middle-ground price. d) Arrange a detailed presentation on value vs. cost. e) Avoid discussing pricing further. **Answer**: b) Firmly explain the value and why the price is justified.

11. COMPETING: **Scenario:** A colleague makes a snide remark about your presentation style, hinting theirs is better. **Options**: a) Agree with them publicly. b) Confidently express the merits of your style, drawing on feedback from others. c) Suggest combining your styles. d) Propose a team training on presentation skills. e) Ignore the remark. **Answer**: b) Confidently express the merits of your style, drawing on feedback from others.

12. COMPETING: **Scenario:** In a review meeting, your supervisor feels your performance is lacking in certain areas. You have data proving otherwise. **Options**: a) Concede to their viewpoint. b) Present the data firmly to challenge their assertion. c) Try to find a middle ground. d) Propose a joint session to delve deeper into performance metrics. e) Avoid discussing it further. **Answer**: b) Present the data firmly to challenge their assertion.

13. COLLABORATION: **Scenario:** Your team member, Mia, feels her ideas are not considered in team decisions and is visibly upset. **Options**: a) Tell her she's overreacting. b) Ignore her feelings and continue the meeting. c) Give in to all her suggestions. d) Arrange a one-on-one to understand her concerns and collaboratively find solutions. e) Propose a blended approach of various team ideas. **Answer**: d) Arrange a one-on-one to understand her concerns and collaboratively find solutions.

14. COLLABORATION: **Scenario:** A client expresses dissatisfaction with the product's user experience. **Options**: a) Defend the product as it is. b) Ignore the feedback and continue the pitch. c) Immediately offer a discount. d) Engage in a collaborative workshop to understand their users' needs and improve the product together. e) Propose a combined feedback session with other clients. **Answer**: d) Engage in a collaborative workshop to understand their users' needs and improve the product together.

15. COLLABORATION: **Scenario:** Your supervisor is anxious about an upcoming project due to past failures. **Options**: a) Tell them it will be fine without much evidence. b) Avoid discussing past failures. c) Give in to whatever approach they suggest. d) Collaboratively review past mistakes and co-create a strategy to ensure success this time. e) Insist on your own way of handling the project. **Answer**: d) Collaboratively review past mistakes and co-create a strategy to ensure success this time.

16. COLLABORATION: **Scenario:** Your co-worker, Alex, is disheartened because his innovative ideas often don't get traction. **Options**: a) Tell him innovation isn't always necessary. b) Avoid discussing his innovative projects. c) Accept all his ideas without critique. d) Collaboratively explore ways to present and implement his innovative ideas effectively. e) Suggest he sticks to standard procedures. **Answer**: d) Collaboratively explore ways to present and implement his innovative ideas effectively.

17. AVOIDANCE: **Scenario:** At a team lunch, two colleagues start a heated argument about a recent project failure. **Options**: a) Intervene and pick a side. b) Start a new topic of conversation to distract them. c) Suggest a compromise for their disagreement. d) Propose a team feedback session post-lunch. e) Excuse yourself from the table without getting involved. **Answer**: e) Excuse yourself from the table without getting involved.

18. AVOIDANCE: **Scenario:** A client sends a scathing email about a minor mistake, blowing it out of proportion. **Options**: a) Reply aggressively defending your team. b) Immediately fix the mistake and ignore the tone of the email. c) Propose a midway solution to pacify them. d) Set up a call to discuss their concerns. e) Wait for a day before addressing the email to let emotions cool. **Answer**: e) Wait for a day before addressing the email to let emotions cool.

19. AVOIDANCE: **Scenario:** Your supervisor, in a bad mood, starts nitpicking minor issues in your report in front of the team. **Options**: a) Defend every point aggressively. b) Agree with every critique to pacify them. c) Suggest a blended approach to the report. d) Ask to discuss the report in a one-on-one setting. e) Stay silent during the critique and revisit the feedback later when the mood is better. **Answer**: e) Stay silent during the critique and revisit the feedback later when the mood is better.

20. AVOIDANCE: **Scenario:** At a conference, a competitor makes a snarky comment about your company's recent product. **Options**: a) Confront them aggressively. b) Publicly praise their company to counteract. c) Propose a combined workshop for mutual growth. d) Engage in a detailed debate about product merits. e) Choose not to engage and focus on networking with other attendees. **Answer**: e) Choose not to engage and focus on networking with other attendees.

I hope these test items meet your requirements! Let me know if you'd like any changes.

PROMPT: Repeat the same 20 items including the response options but expand the scenarios a little bit, leaving the response options and the correct responses unchanged.

RESPONSE: Certainly! Here's an expanded version of the previous scenarios:

1. COMPROMISE: **Scenario:** You and your team member, John, are working on a crucial project. As the deadline approaches, he seems anxious and insists on following his methodology, believing it will be faster. You have your reservations, thinking your approach is more comprehensive and efficient. **Options**:
a) Propose combining elements of both methods.
b) Tell John you will do it your way since you're the senior.
c) Let John decide entirely.
d) Discuss the benefits of both methods in a team meeting.
e) Avoid the conversation and just get the task done as quickly as possible.
Answer: a) Propose combining elements of both methods.

---

2. COMPROMISE: **Scenario:** Lisa, a dedicated coworker, often finds herself double-booked due to meeting overlaps. She expresses her irritation, feeling stretched thin between her commitments and your scheduled meetings. **Options**:
a) Propose alternating meeting times each week.
b) Tell Lisa she should prioritize your meetings.
c) Always adjust to Lisa's schedule.
d) Collaboratively set up a joint calendar to find mutual availability.
e) Don't discuss the clash and hope she attends.
Answer: a) Propose alternating meeting times each week.

---

3. COMPROMISE: **Scenario:** Mr. Smith, a long-term client, feels the latest design you sent over doesn't resonate with his brand vision. He shares a mock-up of his vision, which is significantly different from your team's concept. **Options**:
a) Propose a design that merges both visions.
b) Insist on the original design.
c) Accept Mr. Smith's design completely.
d) Arrange a workshop with Mr. Smith to co-create the design.
e) Avoid any discussion and send the invoice.
Answer: a) Propose a design that merges both visions.

---

4. COMPROMISE: **Scenario:** During a project review, your team member, Rachel, excitedly showcases the outstanding results of her strategy. You're impressed but also notice potential pitfalls that might crop up in the long run. **Options**:
a) Propose tweaks to her strategy to avoid future pitfalls.
b) Demand a change in strategy immediately.
c) Adopt her strategy without question.
d) Set up a brainstorming session to evolve the strategy further.
e) Let it slide and hope the issues never arise.
Answer: a) Propose tweaks to her strategy to avoid future pitfalls.

---

5. ACCOMMODATION: **Scenario:** Your open workspace encourages collaboration, but it has its downsides. Tom, your co-worker who sits adjacent to your desk, is feeling overwhelmed by the noise from your frequent video calls and longs for a quieter workspace. **Options**:
a) Demand he gets noise-cancelling headphones.
b) Offer to take calls in a meeting room to not disturb him.
c) Combine both work patterns.
d) Propose a team discussion about workspace norms.
e) Just continue as normal, hoping he'll adjust.
Answer: b) Offer to take calls in a meeting room to not disturb him.

---

6. ACCOMMODATION: **Scenario:** You've presented a high-risk, high-reward strategy to Mrs. Lopez, a cautious client. Although the projections are promising, she's visibly anxious about potential setbacks and uncertain outcomes. **Options**:
a) Tell her to trust you completely.
b) Switch to a more conservative strategy for her comfort.
c) Seek a middle ground.
d) Offer a joint strategy session to address concerns.

e) Avoid discussing the strategy further.
Answer: b) Switch to a more conservative strategy for her comfort.

---

7. ACCOMMODATION: **Scenario:** During a team huddle, your supervisor questions a decision you made earlier in the week. The tone suggests they're upset about not being consulted, and you can sense the team's unease. **Options**:
a) Defend your decision strongly.
b) Apologize and offer to revert the decision.
c) Find a compromise between your actions and their preference.
d) Propose a feedback session for better decision-making in the future.
e) Avoid discussing it and proceed with other tasks.
Answer: b) Apologize and offer to revert the decision.

---

8. ACCOMMODATION: **Scenario:** At the company's annual gala, everyone is in high spirits. However, a junior colleague is overly ecstatic, drawing attention with boisterous laughter and loud anecdotes, making a few attendees uncomfortable. **Options**:
a) Tell her to calm down immediately.
b) Gently pull her aside and ask if she's okay, letting her express herself.
c) Propose a middle-ground behavior.
d) Organize a team game to channel the excitement.
e) Avoid interacting with her for the evening.
Answer: b) Gently pull her aside and ask if she's okay, letting her express herself.

---

9. COMPETING: **Scenario:** During a team brainstorming session, David, an ambitious team member, openly challenges your expertise. He presents his approach with a confidence that suggests it's vastly superior to yours, causing a few murmurs among the team.
Options:
a) Acknowledge his points and agree.
b) Assert your expertise and experience, clarifying why your approach is right.
c) Propose a blend of both methods.
d) Organize a separate brainstorming session.
e) Ignore his challenge and move on.
Answer: b) Assert your expertise and experience, clarifying why your approach is right.

---

10. COMPETING: **Scenario:** During a contract negotiation, a potential client scrutinizes the pricing breakdown. They lean back, arms crossed, and question if the price is too high given the perceived value. **Options**:
a) Immediately offer a discount.
b) Firmly explain the value and why the price is justified.
c) Propose a middle-ground price.
d) Arrange a detailed presentation on value vs. cost.
e) Avoid discussing pricing further.
Answer: b) Firmly explain the value and why the price is justified.

---

11. COMPETING: **Scenario:** Post a team presentation, during a coffee break, a colleague snidely comments about your delivery, contrasting it with her own refined style. A few peers around you await your reaction.
Options:
a) Agree with them publicly.
b) Confidently express the merits of your style, drawing on feedback from others.
c) Suggest combining your styles.
d) Propose a team training on presentation skills.
e) Ignore the remark.
Answer: b) Confidently express the merits of your style, drawing on feedback from others.

---

12. COMPETING: **Scenario:** In a performance review meeting, your supervisor, equipped with a file of feedback, starts pinpointing areas where they believe your performance fell short. You recall the data and feedback you've collected, which tells a different story. **Options**:
a) Concede to their viewpoint.

b) Present the data firmly to challenge their assertion.
c) Try to find a middle ground.
d) Propose a joint session to delve deeper into feedback.
e) Avoid the confrontation.
Answer: b) Present the data firmly to challenge their assertion.

---

13. COLLABORATION: **Scenario:** Your product launch is around the corner, but product testing has brought forth unforeseen challenges. Your project manager, Sam, feels these issues mirror past mistakes, causing a palpable tension in the team. **Options**:
a) Reassure the team without addressing concerns.
b) Shrug off the comparison with past errors.
c) Comply with whatever approach Sam suggests.
d) Analyze past challenges with Sam and co-develop a robust strategy for the present.
e) Push for your own solutions.
Answer: d) Analyze past challenges with Sam and co-develop a robust strategy for the present.

---

14. COLLABORATION: **Scenario:** Post an ideation workshop, Jenna, a seasoned engineer, feels her ideas often go unnoticed amidst the team's loud discussions. She pulls you aside, frustration evident in her voice. **Options**:
a) Assure her that ideas aren't always about volume.
b) Change the subject and avoid discussing her concerns.
c) Agree to implement her ideas without assessment.
d) Collaboratively design a feedback loop ensuring all ideas get equal attention.
e) Advise her to be more assertive.
Answer: d) Collaboratively design a feedback loop ensuring all ideas get equal attention.

---

15. COLLABORATION: **Scenario:** As the year-end approaches, both finance and marketing departments clash over budget allocations. Lydia, from finance, has her reservations based on past spending patterns, leading to heated debates.
Options:
a) Disregard past spendings, focusing on future gains.
b) Avoid past mistakes' discussion.
c) Concede to finance's budgeting.
d) Engage both departments to collaboratively chart out a mutually agreeable budget plan.
e) Enforce the marketing department's viewpoint.
Answer: d) Engage both departments to collaboratively chart out a mutually agreeable budget plan.

---

16. COLLABORATION: **Scenario:** Your department's latest campaign didn't perform as expected. At a review meeting, Alex, a junior strategist, voices his concern that the team rarely explores non-traditional approaches.
Options:
a) Suggest sticking to tried-and-tested methods.
b) Brush off the need for innovative methods.
c) Accept all of Alex's suggestions.
d) Partner with Alex to incorporate innovative strategies for the next campaign.
e) Recommend Alex attend more training.
Answer: d) Partner with Alex to incorporate innovative strategies for the next campaign.

---

17. AVOIDANCE: **Scenario:** The annual company picnic is in full swing. As everyone enjoys the sunny afternoon, two colleagues, Steve and Maria, begin a heated debate about a client's recent feedback. Some around them try to change the topic, sensing the rising tension. **Options**:
a) Jump in and take a side.
b) Attempt to divert the conversation.
c) Propose a solution for their disagreement.
d) Suggest discussing the feedback once back at the office.
e) Find another group to mingle with, avoiding the brewing storm.
Answer: e) Find another group to mingle with, avoiding the brewing storm.

---

18. AVOIDANCE: **Scenario:** After weeks of hard work, the project is finally ready for delivery. However, an email notification disrupts the celebratory mood. A client, known for their mercurial temperament, has pointed out a trivial error in an abrasive tone. **Options**:
a) Respond with an equally strong defense.
b) Rectify the error immediately without acknowledging the email's tone.
c) Suggest a call to clarify and resolve the issue.
d) Request a face-to-face meeting to discuss.
e) Allow a cooling period before crafting a well-thought-out response.
Answer: e) Allow a cooling period before crafting a well-thought-out response.

---

19. AVOIDANCE: **Scenario:** Monthly report presentations are always stressful. Today, however, your supervisor, known for their mood swings, starts picking apart your slides for insignificant details, all while the team watches in awkward silence. **Options**:
a) Argue each point fiercely.
b) Agree to every critique without argument.
c) Propose a blend of your approach and theirs.
d) Ask to address the points offline, away from the team.
e) Remain composed, making mental notes, and decide to approach them later for clarification.
Answer: e) Remain composed, making mental notes, and decide to approach them later for clarification.

---

20. AVOIDANCE: **Scenario:** The industry conference is the perfect place to network and learn. Yet, as you engage in a lively discussion, a rival company representative throws a veiled jab about your company's newest product line. Other participants shift uncomfortably, awaiting your reaction. **Options**:
a) Retort with a pointed comment about their company.
b) Praise their company's achievements openly.
c) Suggest a collaborative effort for mutual benefit.
d) Enter a detailed debate defending your product.
e) Politely smile and focus on more constructive interactions with other attendees.
Answer: e) Politely smile and focus on more constructive interactions with other attendees.

Supplementary Notes 2: Final ChatGPT-created items

Final ChatGPT-generated items for STEM-B

19. **Emotion: Sadness, Domain: Workplace** Karen's favorite coworker, Sam, has been transferred to another branch, leaving Karen feeling quite lonely. What action would be the most effective for Karen? a) Get upset about Sam's transfer. b) Spend time with other coworkers and build new friendships. (Correct) c) Start looking for a new job. d) Hope that Sam will come back.
20. **Emotion: Anger, Domain: Personal-Life** Ted finds out that his best friend has been spreading rumors about him. What action would be the most effective for Ted? a) Confront his friend and demand an apology. (Correct) b) Cut off all communication with his friend. c) Start spreading rumors about his friend. d) Ignore it and continue as if nothing happened.
21. **Emotion: Fear, Domain: Workplace** Laura is nervous about an upcoming presentation she has to give in front of the company board. What action would be the most effective for Laura? a) Decide not to give the presentation. b) Find someone else to give the presentation. c) Practice the presentation and seek feedback to improve. (Correct) d) Just wing it and hope for the best.
22. **Emotion: Disgust, Domain: Personal-Life** Dave's neighbor's dog keeps leaving messes in his yard. What action would be the most effective for Dave? a) Return the mess to his neighbor's yard. b) Confront his neighbor and express his concern about the issue. (Correct) c) Ignore it and clean up the mess himself. d) Report the neighbor to the police.
23. **Emotion: Sadness, Domain: Personal-Life** Sophia's long-term relationship just ended. What action would be the most effective for Sophia? a) Dwell on the break-up and cry it out. b) Reach out to friends and family for support. (Correct) c) Start dating immediately. d) Ignore her feelings and pretend everything is okay.
24. **Emotion: Anger, Domain: Workplace** Michael's idea was stolen by a colleague, who then received recognition for it. What action would be the most effective for Michael? a) Start a fight with the colleague. b) Talk to his superior about the situation. (Correct) c) Resent his colleague silently. d) Steal an idea from the colleague in return.
25. **Emotion: Fear, Domain: Personal-Life** Lily has been experiencing health problems and is afraid of the doctor's diagnosis. What action would be the most effective for Lily? a) Avoid going to the doctor. b) Try to self-diagnose using the internet. c) Go to the doctor to understand what's happening and discuss her fears. (Correct) d) Ignore her symptoms and hope they will go away.
26. **Emotion: Sadness, Domain: Workplace** Lucy has been passed over for a promotion she was expecting. What action would be the most effective for Lucy? a) Resent her boss for not promoting her. b) Quit her job. c) Seek feedback and work on her professional growth. (Correct) d) Avoid work and give minimal effort.
27. **Emotion: Anger, Domain: Personal-Life** Neil discovered that his brother used his car without asking and damaged it. What action would be the most effective for Neil? a) Refuse to talk to his brother. b) Seek reparations from his brother and discuss the importance of respect for personal property. (Correct) c) Damage his brother's property in retaliation. d) Ignore the damage and let it go.
28. **Emotion: Fear, Domain: Workplace** Olivia fears public speaking and is asked to lead a team meeting. What action would be the most effective for Olivia? a) Refuse to lead the meeting. b) Prepare thoroughly and practice her presentation. (Correct) c) Ask someone else to speak on her behalf. d) Wing it on the day of the meeting.
29. **Emotion: Disgust, Domain: Personal-Life** Max's roommate consistently leaves dirty dishes around the house. What action would be the most effective for Max? a) Start throwing out the dishes. b) Confront his roommate about the issue and suggest a cleaning schedule. (Correct) c) Move out immediately. d) Clean the dishes himself every time.
30. **Emotion: Sadness, Domain: Personal-Life** Julia's child has just moved away for college, leaving her feeling lonely. What action would be the most effective for Julia? a) Try to persuade her child to stay home. b) Find activities she enjoys to fill her time and stay connected with her child. (Correct) c) Sit at home and mourn her loneliness. d) Decide to have another child to replace the one who left.
31. **Emotion: Anger, Domain: Workplace** Ben's supervisor takes credit for his work during a meeting. What action would be the most effective for Ben? a) Publicly accuse his supervisor of stealing his work. b) Discuss the issue privately with his supervisor or report it to higher management. (Correct) c) Do poor work in future projects. d) Quit his job without notice.
32. **Emotion: Fear, Domain: Personal-Life** Susan is anxious about flying and has a trip coming up. What action would be the most effective for Susan? a) Cancel the trip and stay home. b) Learn about the safety of air travel and consider relaxation techniques. (Correct) c) Drink alcohol to calm her nerves. d) Just ignore her fear and hope it will go away.

33. **Emotion: Disgust, Domain: Workplace** Tony is assigned to a project he finds morally objectionable. What action would be the most effective for Tony? a) Do the project anyway. b) Complain about the project to his coworkers. c) Discuss his concerns with his supervisor or consider if the job aligns with his values. (Correct) d) Sabotage the project.

34. **Emotion: Sadness, Domain: Workplace** Anna's team didn't win the company's project competition, which she was very invested in. What action would be the most effective for Anna? a) Blame her team members for the loss. b) Use the experience as a learning opportunity to improve for next time. (Correct) c) Give up participating in future competitions. d) Demand a rematch.

35. **Emotion: Anger, Domain: Personal-Life** Eric found out his spouse forgot their anniversary. What action would be the most effective for Eric? a) Ignore his spouse until they realize what they forgot. b) Express his feelings about the forgotten anniversary and discuss ways to avoid such oversights. (Correct) c) Forget his spouse's birthday in return. d) Divorce his spouse over the oversight.

36. **Emotion: Fear, Domain: Workplace** Rachel is afraid of conflict and her colleagues are having a heated argument. What action would be the most effective for Rachel? a) Avoid the colleagues who are arguing. b) Take a stand and share her viewpoint in a constructive manner. (Correct) c) Take sides and escalate the argument. d) Start a rumor about the colleagues to distract them.

Final ChatGPT-generated items for STEU-B

1. Alex was walking home from work. Something happened that caused him to feel fear. What is most likely to have happened? (a) He remembered he had left his front door unlocked. (b) A group of people were laughing loudly in the distance. (c) A stray dog barked at him but was held back by a fence. (d) He heard footsteps closely following him in a dark alley. ✔ (e) He found out that he left his office keys at his desk.

2. Chris felt a wave of contentment as he gazed at the artwork he had spent weeks perfecting. a) Pride ✔ b) Joy c) Relief d) Surprise e) Gratitude

3. Ryan experienced a pang of discomfort and self-reproach after realizing the hurtful consequences of his remarks on his friend. a) Guilt ✔ b) Shame c) Regret d) Sadness e) Anger

4. Liam’s spirits plummeted as he read the rejection notice from his dream university. a) Sadness b) Regret c) Frustration d) Disappointment ✔ e) Shame

5. Sharon felt an overwhelming sense of warmth as she watched the stranger return her misplaced wallet. a) Relief b) Gratitude ✔ c) Surprise d) Confusion e) Excitement

6. Raul felt a rush of cold air and his heartbeat quicken as he heard footsteps behind him in the dimly lit alley. a) Anxiety b) Fear ✔ c) Surprise d) Distress e) Horror

7. Tom felt waves of annoyance when he discovered his younger brother had again borrowed his laptop without asking. a) Frustration ✔ b) Anger c) Jealousy d) Contempt e) Distress

8. John's spirits lifted when he noticed the first sprout in his garden after weeks of anticipation. a) Surprise b) Hope ✔ c) Excitement d) Joy e) Gratitude

9. Lucia was browsing her emails. Something happened that made her feel excitement. What is most likely to have happened? (a) She saw an email reminding her of a meeting tomorrow. (b) She received an invitation to a weekend getaway with her best friends. ✔ (c) She found an old email from a coworker sharing a recipe. (d) She received a newsletter about a workshop she might attend. (e) She got an email notifying her of a software update.

10. Marco's face turned red as his classmates chuckled at his incorrect response. a) Shame b) Anger c) Confusion d) Distress ✔ e) Frustration

11. Victoria felt a sudden release of tension when she was informed her family was unharmed following the hurricane. a) Relief ✔ b) Gratitude c) Surprise d) Hope e) Excitement

12. Carlos was at a community gathering. Something occurred that made him feel contempt. What is most likely to have happened? (a) He heard someone praising the event organizer. (b) He learned about the charitable activities of the community. (c) Someone spilled a drink on him by accident. (d) He overheard someone rudely and baselessly criticizing his close friend. ✔ (e) He got introduced to a new neighbor.

13. Jenny couldn't keep the smile off her face as she crossed the finish line, feeling elated. a) Excitement b) Joy ✔ c) Pride d) Relief e) Surprise

14. Sarah was reading a letter from her old school. Something in it made her feel ashamed. What is most likely to have happened? (a) The school was inviting alumni for a reunion. (b) The letter described the achievements of her batchmates. (c) She was reminded of an outstanding fee she never paid. ✔ (d) The school asked for donations for a new building. (e) The school was closing down due to financial difficulties.
15. Olivia experienced a sense of fulfillment seeing the bright smiles of the children she had volunteered to assist. a) Gratitude b) Pride c) Joy d) Relief e) Satisfaction ✔
16. Tiffany glared at her neighbor with deep resentment as he played loud music until the early hours. a) Anger ✔ b) Frustration c) Distress d) Contempt e) Jealousy
17. Nina watched as her younger sibling received a trophy for remarkable achievements in science. a) Jealousy b) Pride ✔ c) Contempt d) Excitement e) Surprise
18. As Leo noticed the abandoned puppy in the rain, he felt a profound desire to provide it with shelter. a) Sadness b) Compassion c) Relief d) Distress ✔ e) Regret
19. Sophia's chest tightened and her eyes welled up as she reached the concluding passages of the poignant novel. a) Sadness ✔ b) Distress c) Regret d) Confusion e) Surprise

Final ChatGPT-generated items for GEMOK Blends

1. **Anna attends her school reunion.** The room buzzes with nostalgic laughter and chatter. As she moves to get a drink, she overhears some old classmates gathered in a corner. They discuss her recent success as an author. They don't realize she's nearby. One mentions how they never expected Anna to do so well, while another admits being impressed by her achievements.
   - Pride and irritation
   - Joy and anger
   - Surprise and happiness
   - Interest and contempt
   - Sadness and pleasure

   Solution: Pride and irritation

2. **Ben watches his son's piano recital.** The auditorium is filled with expectant parents and well-wishers. His son begins with confidence but stumbles on some notes midway. Ben's heart skips a beat, memories of his own failures as a musician flooding back. However, his son regains composure, finishing with a flourish, earning applause from the audience.
   - Sadness and pride
   - Anger and interest
   - Joy and disgust
   - Fear and pleasure
   - Anxiety and happiness

   Solution: Sadness and pride

3. **Clara discovers a secret letter tucked in a drawer.** The handwriting belongs to her partner. As she reads through, the content mentions another person. But, as she delves deeper, she realizes it's about organizing a surprise birthday party for her. The intricate planning details reflect the enormous effort her partner has invested.
   - Joy and jealousy
   - Surprise and guilt
   - Interest and anxiety
   - Happiness and anger
   - Pleasure and irritation

   Solution: Surprise and guilt

4. **David, a dedicated teacher, sits in his study reviewing exam papers.** The room is silent except for the rustle of pages. An exceptionally well-written essay catches his attention. Yet, as he reads on, he identifies familiar phrases from a renowned publication. The realization that one of his brightest students might have plagiarized makes him pause.
   - Interest and anger
   - Joy and disgust

- Shame and happiness
- Sadness and pride
- Contempt and anxiety
  Solution: Contempt and anxiety

5. **Emma casually flips through channels on a lazy afternoon.** She stops abruptly when she recognizes a familiar face. It's her childhood friend, now a famous actress, being interviewed. Emma recalls their shared dreams of fame, and while she opted for a different life, her friend chased the limelight. As her friend thanks her parents in a speech, Emma's past and present collide.
- Jealousy and joy
- Sadness and pride
- Interest and anger
- Happiness and irritation
- Fear and pleasure
  Solution: Jealousy and joy

6. **Frank, an artist, attends a friend's art exhibition downtown.** The gallery is filled with art enthusiasts. While moving from one piece to another, Frank's eyes fall on a painting that seems strikingly similar to a creation he made years ago. As the evening progresses, the painting garners considerable attention and compliments, leaving Frank in introspection.
- Pride and disgust
- Interest and anxiety
- Sadness and joy
- Anger and pleasure
- Contempt and happiness
  Solution: Contempt and happiness

7. **Grace, while cleaning the attic, finds an old, dusty journal.** The cover indicates it belonged to her mother. Flipping through the pages, Grace discovers struggles and challenges her mother faced but never vocalized. Each entry gives Grace a deeper understanding of the sacrifices her mother made.
- Sadness and interest
- Joy and surprise
- Pride and anxiety
- Happiness and fear
- Anger and pleasure
  Solution: Sadness and interest

8. **Henry, immersed in work, receives an unexpected email notification.** The subject suggests a postponed project approval, which he had been eagerly awaiting. He realizes this delay could jeopardize his upcoming promotion. Yet, later in the day, he discovers the postponement was due to a senior colleague wanting to include Henry's innovative ideas for a broader implementation.
- Surprise and irritation
- Interest and anger
- Joy and jealousy
- Anxiety and pride
- Fear and happiness
  Solution: Anxiety and pride

9. **Isabelle's morning coffee is interrupted by a sensational news headline on TV.** Her brother's name flashes in bold. A lump forms in her throat, but as she listens, she learns it's a positive story about him preventing a scandalous event, despite the misleading headline.
- Anger and pride
- Joy and disgust
- Fear and interest
- Surprise and sadness
- Pleasure and anxiety
  Solution: Anger and pride

10. **Jack sits on his porch, sipping tea as he listens to his daughter practicing her speech indoors.** The pauses, retries, and stumbles remind him of his own challenges growing up. Her relentless dedication, despite the mistakes, takes him on a trip down memory lane.
- Sadness and joy

- Pride and fear
- Surprise and irritation
- Disgust and happiness
- Interest and anger

  Solution: Sadness and joy

11. **Karen, arriving home after a tiring day, spots a package at her doorstep.** With no sender's name, she curiously unwraps it. Inside is a cherished book from her childhood, bringing back a flood of memories, some pleasant and others tinged with pain.
- Surprise and sadness
- Joy and fear
- Pride and anxiety
- Happiness and contempt
- Disgust and interest

  Solution: Surprise and sadness

12. **Liam, on a quiet evening, decides to watch a documentary.** The gripping real-life stories about a pressing global issue deeply move him. He's affected not just by the narratives but also by the raw emotions displayed, making him contemplate his role in the larger scheme of things.
- Sadness and interest
- Anger and pleasure
- Joy and anxiety
- Fear and pride
- Disgust and happiness

  Solution: Sadness and interest

13. **Monica, donning a beautiful bridesmaid dress, attends her best friend's wedding.** As she watches her friend walk down the aisle, memories of their childhood games and pretend weddings flash before her eyes. Their innocent promises of the past juxtapose with the reality of the present.
- Joy and jealousy
- Sadness and pride
- Happiness and fear
- Interest and disgust
- Surprise and contempt

  Solution: Joy and jealousy

14. **Nathan, out on his morning walk, discovers a lost puppy whimpering near his house.** He takes the initiative to find its owner, putting up posters and posts on social media. When the owner finally arrives, Nathan's taken aback — it's someone from his past who had once wronged him.
- Disgust and interest
- Anger and pleasure
- Fear and joy
- Shame and happiness
- Sadness and pride

  Solution: Disgust and interest

15. **Olivia, engrossed in her work, is startled by a ping on her computer.** It's feedback from her boss about the project she submitted last week. The initial praises make her heart swell, but as she reads further, she's made aware of some overlooked errors that might have serious implications.
- Pride and anxiety
- Joy and fear
- Anger and happiness
- Surprise and sadness
- Disgust and interest

  Solution: Pride and anxiety

16. **Peter, on a serene evening, receives an elegant invitation card.** The glossy card mentions an exclusive event in the city's most upscale venue. He's thrilled but while preparing, a call informs him the invitation was sent by mistake and was intended for someone else with a similar name.
- Surprise and sadness
- Anger and pride

- Interest and disgust
- Fear and joy
- Shame and pleasure

  Solution: Shame and pleasure

17. **Quinn, while browsing her social media feed, stumbles upon a post discussing her recent achievements.** The post, dripping with sarcasm, mocks her efforts. However, scrolling through the comments, she discovers an army of strangers ardently defending and praising her accomplishments.
    - Anger and pride
    - Joy and jealousy
    - Surprise and fear
    - Sadness and happiness
    - Disgust and interest

    Solution: Anger and pride

18. **Rachel, during a summer visit to her grandparents' old house, stumbles upon a stack of old photos.** One particular photo catches her eye — her grandfather, much younger, receiving a prestigious award. The image paints a picture of a side of him she never knew, making her want to delve deeper into his past.
    - Interest and joy
    - Sadness and pride
    - Surprise and contempt
    - Happiness and disgust
    - Fear and pleasure

    Solution: Interest and joy

19. **Steve, eager to improve his health, joins a month-long fitness challenge at work.** Days of rigorous training culminate in a finale event. Despite giving his best, he finishes second. But the winner, during the award ceremony, candidly admits being inspired by Steve's unwavering dedication throughout the challenge.
    - Sadness and pride
    - Disgust and happiness
    - Surprise and anxiety
    - Fear and interest
    - Anger and joy

    Solution: Sadness and pride

20. **Tara, after a long phone call, walks into her living room to find her child busy with crayons.** The walls, previously pristine, now sport vibrant, albeit messy, drawings. While the initial shock is overwhelming, she can't help but notice the vivid imagination her child has showcased in every scribble.
    - Anger and pleasure
    - Disgust and pride
    - Fear and interest
    - Surprise and sadness
    - Joy and contempt

    Solution: Disgust and pride

Final ChatGPT-generated items for GECo Emotion Regulation

Note: The strategies in parentheses (e.g., rumination) are not displayed to participants. Participants are asked to select two out of the four options. For each adaptive strategy option selected, award 0.5 points (for each item, a maximum score of 1 can be reached). Adaptive strategies are positive reappraisal, acceptance, refocusing on planning, putting into perspective, positive refocusing.

7. **After spending weeks preparing a detailed proposal, you're taken aback to learn that it was overshadowed by a simpler idea from a colleague. This sudden revelation fills you with irritation.** a. You consider the feedback for future improvements. (positive reappraisal) b. You ponder why management always favors others. (rumination) c. You decide to discuss with your colleague for a combined approach next time. (refocusing on planning) d. You blame yourself for not presenting it better. (blaming oneself)
8. **An unforeseen complication arises in your project. The initial timeline, which was already tight, now seems impossible. Waves of anxiety rush over you as the looming deadline approaches.** a. You strategize a new approach to make up for lost time. (refocusing on planning) b. You think it's always your luck that projects get delayed. (catastrophizing) c. You see it as an opportunity to refine the final output. (positive refocusing) d. You dwell on every small mistake that led to this. (rumination)
9. **While taking a break, you accidentally overhear a couple of colleagues in the next room discussing your recent error in detail. The weight of their words bears down on you, immersing you in sadness.** a. You decide to address the error in the next team meeting. (acceptance) b. You think they always find a reason to talk behind your back. (blaming others) c. You take a moment to think of your past successes. (positive refocusing) d. You constantly replay their words in your mind. (rumination)
10. **You had been planning a family vacation for months. When you finally muster the courage to apply for leave, it gets declined due to some organizational priorities. The frustration is palpable.** a. You reevaluate the importance of taking those specific days off. (putting into perspective) b. You blame HR for always being so inflexible. (blaming others) c. You think of alternative dates to apply for leave. (refocusing on planning) d. You feel this always happens when you really need a break. (catastrophizing)
11. **Every year, your team has a chance to pitch new initiatives. This year, you had a groundbreaking idea, but upon presenting, it's met with disinterest. Anger bubbles up as you consider the hours of research and preparation.** a. You decide to seek feedback and refine the idea for next time. (refocusing on planning) b. You assume the team never really values your suggestions. (blaming others) c. You take solace in the fact that not every idea will be a hit and remain optimistic. (positive refocusing) d. You replay the presentation, pinpointing moments of potential failure. (rumination)
12. **The department undergoes an audit. While you've been diligent, a minor oversight from months ago resurfaces, drawing unwanted attention. The scrutiny and accompanying anxiety are almost paralyzing.** a. You recognize the need for stringent checks in the future. (acceptance) b. You can't shake off the feeling that someone intentionally highlighted the oversight. (blaming others) c. You decide to develop a checklist to avoid such lapses in the future. (refocusing on planning) d. You're consumed by the thought of what other errors might come to light. (catastrophizing)
13. **You've been working tirelessly on a project for months. Just as it's about to launch, management pulls the plug, citing shifting priorities. The disappointment is crushing.** a. You reflect on the learnings you've gained from the project regardless of its outcome. (positive reappraisal) b. You blame the top management for not recognizing the project's value. (blaming others) c. You think about how to repurpose your work for future projects. (refocusing on planning) d. You keep questioning your worthiness in the organization. (blaming oneself)
14. **Every team member was assigned a challenging client. Yours seems particularly difficult, always raising last-minute issues. Their demands keep you on your toes, constantly inducing fear.** a. You analyze their feedback to see if there's a pattern to anticipate future concerns. (refocusing on planning) b. You feel like you're always the one stuck with the problematic clients. (catastrophizing) c. You acknowledge that challenging clients can offer valuable lessons. (acceptance) d. You can't help but think others secretly enjoy watching you struggle. (blaming others)
15. **You've recently been promoted. With new responsibilities, you find yourself struggling to keep up. Colleagues you once considered friends seem distant, making you feel isolated and sad.** a. You take a step back to consider that everyone goes through adjustment phases. (putting into perspective) b. You start doubting if the promotion was a setup for failure. (rumination) c. You decide to attend a training or workshop to hone your skills for the new role. (positive refocusing) d. You blame yourself for not living up to the new expectations. (blaming oneself)
16. **Despite all your efforts, your department's monthly performance has been lagging for the third consecutive month. The mounting pressure and inevitable confrontations fill you with anxiety.** a. You strategize with your team on ways to pivot and make improvements. (refocusing on planning) b. You find yourself replaying past decisions, wondering where you went wrong. (rumination) c. You focus on the small wins and celebrate them to build morale.

(positive refocusing) d. You can't shake off the belief that someone in the team might be sabotaging efforts. (blaming others)

17. **You present a new, innovative approach to a recurring problem in a meeting. However, it's met with immediate resistance from a senior member, causing public embarrassment and anger.** a. You seek private feedback to understand their concerns better. (positive reappraisal) b. You dwell on how your ideas are never given a fair chance. (catastrophizing) c. You remind yourself of the importance of diverse perspectives in refining ideas. (putting into perspective) d. You resentfully blame the senior member for always being stuck in their ways. (blaming others)
18. **An error in your financial reports gets highlighted during a review. It's a minor mistake but has big implications. The weight of this mistake drags you into despair.** a. You acknowledge the mistake and focus on implementing preventive measures. (acceptance) b. You keep pondering how you could be so careless. (blaming oneself) c. You decide to seek mentorship or training in financial reporting. (positive refocusing) d. You feel like these mistakes define your entire career. (catastrophizing)
19. **You've been leading a team virtually due to the current work-from-home scenario. The lack of physical interaction has its challenges, and you fear miscommunication and its repercussions.** a. You think of organizing virtual team-building exercises to enhance cohesion. (refocusing on planning) b. You start to believe that remote work is inevitably doomed to fail. (catastrophizing) c. You remind yourself that every new mode of working has its learning curve. (positive reappraisal) d. You blame the organization for not providing adequate training for remote work. (blaming others)
20. **A client sends a harsh email criticizing your team's work. You can't help but feel angered and questioned about your team's competency.** a. You consider discussing the feedback with the client to ensure future alignment. (refocusing on planning) b. You feel like your team is always at the receiving end of undue criticism. (catastrophizing) c. You evaluate the feedback to extract constructive points. (positive reappraisal) d. You blame a specific team member for not catching the issue earlier. (blaming others)
21. **You missed an important deadline, leading to a delay in project delivery. The oversight fills you with anxiety, anticipating the client's reaction.** a. You accept the oversight and think of ways to prevent it in the future. (acceptance) b. You can't stop ruminating over the consequences of this delay. (rumination) c. You brainstorm with your team about damage control and possible solutions. (positive refocusing) d. You blame yourself endlessly for not being more attentive. (blaming oneself)
22. **During a video call, a colleague unexpectedly challenges your solution. Their unexpected opposition throws you into a state of irritation.** a. You appreciate diverse viewpoints and plan a follow-up discussion. (positive reappraisal) b. You think they are intentionally trying to undermine you. (blaming others) c. You re-evaluate your proposal considering their points. (refocusing on planning) d. You feel like maybe you're not cut out for these kinds of discussions. (blaming oneself)
23. **Despite your hard work, another colleague gets the credit for a project's success. The oversight leaves you feeling overlooked and filled with despair.** a. You decide to communicate more proactively about your contributions in the future. (refocusing on planning) b. You ruminate about how your efforts always go unnoticed. (rumination) c. You focus on the project's success rather than who got the credit. (positive refocusing) d. You feel the management always favors certain individuals. (blaming others)
24. **You receive feedback that your presentation style lacks energy and enthusiasm. This catches you off-guard and fills you with sadness.** a. You see it as an opportunity to improve and consider joining a public speaking course. (positive refocusing) b. You believe that no matter what you do, it will never be enough. (catastrophizing) c. You reflect on previous presentations to understand this feedback better. (positive reappraisal) d. You blame yourself for not being naturally charismatic. (blaming oneself)
25. **A project you were passionate about gets discontinued due to budget cuts. The decision is disheartening and stirs feelings of anger.** a. You remind yourself of the broader business context and its challenges. (putting into perspective) b. You can't stop thinking about the wasted time and effort. (rumination) c. You think of ways to adapt the work done into another valuable initiative. (refocusing on planning) d. You believe that management doesn't value your contributions. (blaming others)
26. **You're assigned a new team member who frequently disagrees with your approaches. The constant disagreements induce feelings of anxiety.** a. You recognize the value of diverse perspectives in achieving optimal solutions. (acceptance) b. You feel the new team member is intentionally trying to sabotage your efforts. (blaming others) c. You consider scheduling regular check-ins to align on project objectives. (refocusing on planning) d. You start doubting your capabilities as a leader. (blaming oneself)
27. **While leading a workshop, technical glitches disrupt the flow, leaving participants visibly frustrated. You feel a rising tide of anxiety about the potential fallout.** a. You acknowledge the hiccups and quickly switch to an alternative discussion until the issues are resolved. (refocusing on planning) b. You believe you should've foreseen and prevented

every technical problem. (blaming oneself) c. You consider this an opportunity to showcase adaptability under pressure. (positive reappraisal) d. You continuously replay the attendees' irritated faces in your mind. (rumination)

28. **During a company retreat, you overhear colleagues discussing your recent project in a critical tone. The covert criticism fills you with sadness and anger.** a. You decide to approach them later for direct feedback to understand their concerns. (positive refocusing) b. You're certain that they've always had a vendetta against you. (blaming others) c. You remind yourself that everyone is entitled to their opinions, and it's essential to separate work from personal feelings. (putting into perspective) d. You fear this might be the general opinion behind your back. (catastrophizing)
29. **A promotion you had been eyeing goes to a less experienced colleague. This unexpected decision instills feelings of despair and confusion.** a. You seek feedback on areas of improvement and focus on your professional growth. (refocusing on planning) b. You think of the countless evenings you spent working late and feel it's all been in vain. (rumination) c. You congratulate your colleague, genuinely hoping they excel in the role. (acceptance) d. You're sure there's office politics at play here. (blaming others)
30. **Your proposal for a new initiative gets declined without much explanation. The abrupt dismissal leaves you feeling anxious and uncertain about your standing.** a. You take a step back and reflect on potential areas in your proposal that could be strengthened. (positive reappraisal) b. You constantly worry about how this rejection will impact your reputation. (rumination) c. You consult mentors and peers to refine your idea for a future pitch. (refocusing on planning) d. You believe decision-makers intentionally sideline your suggestions. (blaming others)
31. **Despite your best efforts, a client decides to end their contract early. This unforeseen setback fills you with anger and desolation.** a. You conduct a thorough review to learn from this experience for future client relationships. (positive refocusing) b. You are convinced the client had unrealistic expectations from the get-go. (blaming others) c. You remind yourself of the volatile nature of the business world and use this as a stepping stone. (putting into perspective) d. You feel that maybe you're not cut out for this role. (blaming oneself)
32. **During a team-building activity, your idea gets overlooked in favor of another, making you feel insignificant and undervalued.** a. You respect the group's decision and actively participate in executing the chosen idea. (acceptance) b. You assume your team doesn't value your input. (catastrophizing) c. You think of ways to communicate your ideas more compellingly in future discussions. (refocusing on planning) d. You can't stop thinking about how your idea might have been better. (rumination)
33. **While presenting a quarterly report, you mistakenly share outdated figures, leading to a wave of confusion. The blunder brings feelings of intense embarrassment and fear.** a. You immediately correct the oversight and apologize for the confusion, ensuring accuracy in the future. (positive refocusing) b. You feel that such mistakes might cost you your job. (catastrophizing) c. You consider this a learning curve and think of implementing a verification process. (positive reappraisal) d. You blame the data team for not updating the figures timely. (blaming others)
34. **After a year of hard work, the product you've been developing receives mixed reviews post-launch. The criticism makes you question the entire project's worth.** a. You delve into the feedback, identifying areas that can be enhanced in subsequent versions. (refocusing on planning) b. You regret the time spent on this project, feeling it was all a waste. (rumination) c. You remind yourself of the innovation the product brings and its potential with some tweaks. (positive reappraisal) d. You believe certain team members didn't put in their best, leading to this outcome. (blaming others)

Final ChatGPT-generated items for GECo Emotion Management

1. You and your team member, John, are working on a crucial project. As the deadline approaches, he seems anxious and insists on following his methodology, believing it will be faster. You have your reservations, thinking your approach is more comprehensive and efficient. **Options**:

a) Propose combining elements of both methods.
b) Tell John you will do it your way since you're the senior.
c) Let John decide entirely.
d) Discuss the benefits of both methods in a team meeting.
e) Avoid the conversation and just get the task done as quickly as possible.
Correct answer: a) Propose combining elements of both methods.

2. Lisa, a dedicated coworker, often finds herself double-booked due to meeting overlaps. She expresses her irritation, feeling stretched thin between her commitments and your scheduled meetings. **Options**:

a) Propose alternating meeting times each week.
b) Tell Lisa she should prioritize your meetings.
c) Always adjust to Lisa's schedule.
d) Collaboratively set up a joint calendar to find mutual availability.

e) Don't discuss the clash and hope she attends.
Correct answer: a) Propose alternating meeting times each week.

3. Mr. Smith, a long-term client, feels the latest design you sent over doesn't resonate with his brand vision. He shares a mock-up of his vision, which is significantly different from your team's concept. **Options**:

a) Propose a design that merges both visions.
b) Insist on the original design.
c) Accept Mr. Smith's design completely.
d) Arrange a workshop with Mr. Smith to co-create the design.
e) Avoid any discussion and send the invoice.
Correct answer: a) Propose a design that merges both visions.

4. During a project review, your team member, Rachel, excitedly showcases the outstanding results of her strategy. You're impressed but also notice potential pitfalls that might crop up in the long run. **Options**:

a) Propose tweaks to her strategy to avoid future pitfalls.
b) Demand a change in strategy immediately.
c) Adopt her strategy without question.
d) Set up a brainstorming session to evolve the strategy further.
e) Let it slide and hope the issues never arise.
Correct answer: a) Propose tweaks to her strategy to avoid future pitfalls.

5. Your open workspace encourages collaboration, but it has its downsides. Tom, your co-worker who sits adjacent to your desk, is feeling overwhelmed by the noise from your frequent video calls and longs for a quieter workspace. **Options**:

a) Demand he gets noise-cancelling headphones.
b) Offer to take calls in a meeting room to not disturb him.
c) Combine both work patterns.
d) Propose a team discussion about workspace norms.
e) Just continue as normal, hoping he'll adjust.
Correct answer: b) Offer to take calls in a meeting room to not disturb him.

6. You've presented a high-risk, high-reward strategy to Mrs. Lopez, a cautious client. Although the projections are promising, she's visibly anxious about potential setbacks and uncertain outcomes. **Options**:

a) Tell her to trust you completely.
b) Switch to a more conservative strategy for her comfort.
c) Seek a middle ground.
d) Offer a joint strategy session to address concerns.
e) Avoid discussing the strategy further.
Correct answer: b) Switch to a more conservative strategy for her comfort.

7. During a team huddle, your supervisor questions a decision you made earlier in the week. The tone suggests they're upset about not being consulted, and you can sense the team's unease. **Options**:

a) Defend your decision strongly.
b) Apologize and offer to revert the decision.
c) Find a compromise between your actions and their preference.
d) Propose a feedback session for better decision-making in the future.
e) Avoid discussing it and proceed with other tasks.
Correct answer: b) Apologize and offer to revert the decision.

8. At the company's annual gala, everyone is in high spirits. However, a junior colleague is overly ecstatic, drawing attention with boisterous laughter and loud anecdotes, making a few attendees uncomfortable. **Options**:

a) Tell her to calm down immediately.
b) Gently pull her aside and ask if she's okay, letting her express herself.
c) Propose a middle-ground behavior.
d) Organize a team game to channel the excitement.
e) Avoid interacting with her for the evening.
Correct answer: b) Gently pull her aside and ask if she's okay, letting her express herself.

9. During a team brainstorming session, David, an ambitious team member, openly challenges your expertise. He presents his approach with a confidence that suggests it's vastly superior to yours, causing a few murmurs among the team. **Options**:

a) Acknowledge his points and agree.
b) Assert your expertise and experience, clarifying why your approach is right.
c) Propose a blend of both methods.
d) Organize a separate brainstorming session.
e) Ignore his challenge and move on.
Correct answer: b) Assert your expertise and experience, clarifying why your approach is right.

10. During a contract negotiation, a potential client scrutinizes the pricing breakdown. They lean back, arms crossed, and question if the price is too high given the perceived value. **Options**:

a) Immediately offer a discount.
b) Firmly explain the value and why the price is justified.
c) Propose a middle-ground price.
d) Arrange a detailed presentation on value vs. cost.
e) Avoid discussing pricing further.
Correct answer: b) Firmly explain the value and why the price is justified.

11. Post a team presentation, during a coffee break, a colleague snidely comments about your delivery, contrasting it with her own refined style. A few peers around you await your reaction. **Options**:

a) Agree with them publicly.
b) Confidently express the merits of your style, drawing on feedback from others.
c) Suggest combining your styles.
d) Propose a team training on presentation skills.
e) Ignore the remark.
Correct answer: b) Confidently express the merits of your style, drawing on feedback from others.

12. In a performance review meeting, your supervisor, equipped with a file of feedback, starts pinpointing areas where they believe your performance fell short. You recall the data and feedback you've collected, which tells a different story. **Options**:

a) Concede to their viewpoint.
b) Present the data firmly to challenge their assertion.
c) Try to find a middle ground.
d) Propose a joint session to delve deeper into feedback.
e) Avoid the confrontation.
Correct answer: b) Present the data firmly to challenge their assertion.

13. Your product launch is around the corner, but product testing has brought forth unforeseen challenges. Your project manager, Sam, feels these issues mirror past mistakes, causing a palpable tension in the team. **Options**:

a) Reassure the team without addressing concerns.
b) Shrug off the comparison with past errors.
c) Comply with whatever approach Sam suggests.
d) Analyze past challenges with Sam and co-develop a robust strategy for the present.
e) Push for your own solutions.
Correct answer: d) Analyze past challenges with Sam and co-develop a robust strategy for the present.

14. Post an ideation workshop, Jenna, a seasoned engineer, feels her ideas often go unnoticed amidst the team's loud discussions. She pulls you aside, frustration evident in her voice. **Options**:

a) Assure her that ideas aren't always about volume.
b) Change the subject and avoid discussing her concerns.
c) Agree to implement her ideas without assessment.
d) Collaboratively design a feedback loop ensuring all ideas get equal attention.
e) Advise her to be more assertive.
Correct answer: d) Collaboratively design a feedback loop ensuring all ideas get equal attention.

15. As the year-end approaches, both finance and marketing departments clash over budget allocations. Lydia, from finance, has her reservations based on past spending patterns, leading to heated debates. **Options**:

a) Disregard past spendings, focusing on future gains.
b) Avoid past mistakes' discussion.
c) Concede to finance's budgeting.
d) Engage both departments to collaboratively chart out a mutually agreeable budget plan.
e) Enforce the marketing department's viewpoint.
Correct answer: d) Engage both departments to collaboratively chart out a mutually agreeable budget plan.

16. Your department's latest campaign didn't perform as expected. At a review meeting, Alex, a junior strategist, voices his concern that the team rarely explores non-traditional approaches. **Options**:

a) Suggest sticking to tried-and-tested methods.
b) Brush off the need for innovative methods.
c) Accept all of Alex's suggestions.
d) Partner with Alex to incorporate innovative strategies for the next campaign.
e) Recommend Alex attend more training.
Correct answer: d) Partner with Alex to incorporate innovative strategies for the next campaign.

17. The annual company picnic is in full swing. As everyone enjoys the sunny afternoon, two colleagues, Steve and Maria, begin a heated debate about a client's recent feedback. Some around them try to change the topic, sensing the rising tension. **Options**:

a) Jump in and take a side.
b) Attempt to divert the conversation.
c) Propose a solution for their disagreement.
d) Suggest discussing the feedback once back at the office.
e) Find another group to mingle with, avoiding the brewing storm.
Correct answer: e) Find another group to mingle with, avoiding the brewing storm.

18. After weeks of hard work, the project is finally ready for delivery. However, an email notification disrupts the celebratory mood. A client, known for their mercurial temperament, has pointed out a trivial error in an abrasive tone. **Options**:

a) Respond with an equally strong defense.
b) Rectify the error immediately without acknowledging the email's tone.
c) Suggest a call to clarify and resolve the issue.
d) Request a face-to-face meeting to discuss.
e) Allow a cooling period before crafting a well-thought-out response.
Correct answer: e) Allow a cooling period before crafting a well-thought-out response.

19. Monthly report presentations are always stressful. Today, however, your supervisor, known for their mood swings, starts picking apart your slides for insignificant details, all while the team watches in awkward silence. **Options**:

a) Argue each point fiercely.
b) Agree to every critique without argument.
c) Propose a blend of your approach and theirs.
d) Ask to address the points offline, away from the team.
e) Remain composed, making mental notes, and decide to approach them later for clarification.
Correct answer: e) Remain composed, making mental notes, and decide to approach them later for clarification.

20. The industry conference is the perfect place to network and learn. Yet, as you engage in a lively discussion, a rival company representative throws a veiled jab about your company's newest product line. Other participants shift uncomfortably, awaiting your reaction. **Options**:

a) Retort with a pointed comment about their company.
b) Praise their company's achievements openly.
c) Suggest a collaborative effort for mutual benefit.
d) Enter a detailed debate defending your product.
e) Politely smile and focus on more constructive interactions with other attendees.
Correct answer: e) Politely smile and focus on more constructive interactions with other attendees.

Supplementary Notes 3: Similarity rating study full results

Supplementary Table 1

Average similarity ratings for all combinations between original STEU scenarios and ChatGPT-created STEU scenarios; part 1 (original items 1-22)

		**ChatGPT-created items**																		
		**1**	**2**	**3**	**4**	**5**	**6**	**7**	**8**	**9**	**10**	**11**	**12**	**13**	**14**	**15**	**16**	**17**	**18**	**19**
**Original test items**	**1**	3.1	1.4	1.7	2.7	1.0	2.0	2.4	1.6	1.1	2.7	1.4	2.8	2.1	1.4	1.6	1.6	1.1	1.5	2.6
	**2**	1.0	3.3	1.0	1.1	1.9	1.1	1.0	2.2	3.3	1.6	1.6	1.1	3.3	1.2	2.9	1.0	1.4	1.3	3.0
	**3**	1.3	2.0	1.0	1.0	1.6	1.3	1.1	2.7	2.0	1.3	2.7	1.7	2.7	1.0	1.0	3.4	1.0	1.3	1.3
	**4**	1.3	1.9	1.0	1.0	1.6	1.3	1.1	2.7	2.5	1.0	3.4	2.1	2.9	1.0	1.1	1.0	1.1	1.6	2.6
	**5**	1.3	1.0	3.6	2.4	1.0	2.1	2.0	1.0	1.0	1.4	1.8	1.7	1.0	3.0	1.1	2.1	1.3	1.4	1.7
	**6**	1.0	1.7	1.3	1.4	1.4	1.1	1.0	1.9	2.2	1.0	1.3	2.0	1.4	1.0	2.0	1.0	2.0	1.6	1.4
	**7**	3.4	1.3	3.1	4.8	1.0	4.3	2.5	1.0	1.2	2.9	1.9	4.1	1.1	1.9	1.1	3.2	1.1	1.4	1.7
	**8**	1.3	1.7	1.3	1.3	1.7	1.1	1.0	2.0	3.0	1.1	2.7	1.9	3.9	1.1	2.3	1.0	2.3	2.0	2.0
	**9**	1.9	1.1	2.1	2.3	1.6	2.0	4.6	1.1	1.1	1.4	1.1	2.7	1.1	1.9	1.1	1.9	2.0	1.9	1.6
	**10**	2.3	2.0	1.7	2.5	2.4	3.6	1.1	2.8	2.5	1.5	2.1	1.7	3.0	1.5	1.3	1.5	2.0	1.1	2.6
	**11**	1.7	1.5	1.3	2.9	1.0	2.3	1.9	1.0	1.3	2.9	1.4	2.3	1.0	2.9	1.6	2.1	2.2	1.4	1.1
	**12**	2.6	1.0	1.9	2.0	1.3	1.0	3.4	1.3	1.0	2.1	1.0	3.1	1.3	2.0	1.0	2.7	1.0	1.4	1.5
	**13**	2.0	1.3	1.4	2.6	1.0	2.5	1.1	1.1	1.3	1.6	1.9	1.6	1.4	1.7	1.0	1.4	1.4	1.1	2.0
	**14**	4.0	1.0	2.6	2.9	1.0	1.9	3.7	1.5	1.0	1.9	1.3	2.4	1.0	2.1	1.0	2.6	1.0	1.1	1.1
	**15**	2.0	1.3	1.9	2.2	1.2	1.1	2.3	1.1	1.4	1.6	1.0	1.9	1.0	1.6	1.1	2.3	1.6	1.1	1.0
	**16**	1.6	1.1	1.0	3.0	1.0	1.0	1.7	1.0	1.0	1.0	1.4	2.8	1.6	1.0	1.7	1.1	1.0	1.6	1.3
	**17**	1.7	1.9	1.0	1.1	1.4	1.3	1.0	2.7	2.6	1.0	1.7	1.3	2.0	2.0	1.7	1.0	2.1	1.9	2.0
	**18**	1.1	1.0	2.3	2.0	1.0	1.4	1.5	1.3	1.3	1.9	1.0	3.0	1.1	1.6	1.1	3.3	1.3	1.0	1.3
	**19**	2.6	1.3	2.3	2.6	1.4	1.6	1.7	1.0	2.4	1.1	1.3	2.1	1.0	2.8	1.0	2.0	1.6	1.1	1.4
	**20**	4.2	1.7	1.1	1.9	2.2	2.9	1.6	2.4	4.1	2.0	1.6	2.6	1.1	1.7	2.0	1.1	1.7	1.3	2.7
	**21**	2.6	1.4	1.9	2.0	1.0	1.8	1.6	1.0	1.0	3.0	1.4	2.1	1.0	1.4	1.0	1.7	1.1	1.5	1.4
	**22**	1.0	2.3	1.3	1.4	5.0	1.0	1.3	2.3	3.3	1.0	2.7	1.1	1.7	1.4	3.1	1.8	4.0	1.4	1.1

Note. Scenario pairs were rated on a 7-point scale from 1 = very different to 7 = very similar. Average ratings of 5.0 and higher are highlighted in blue.

Supplementary Table 2

Average similarity ratings for all combinations between original STEU scenarios and ChatGPT-created STEU scenarios; part 2 (original items 23-42)

		**ChatGPT-created items**																		
		**1**	**2**	**3**	**4**	**5**	**6**	**7**	**8**	**9**	**10**	**11**	**12**	**13**	**14**	**15**	**16**	**17**	**18**	**19**
**Original test items**	**23**	1.4	1.7	1.1	1.0	2.1	1.0	1.0	2.5	5.0	1.1	1.0	2.3	4.1	1.1	3.7	1.1	3.8	1.3	1.3
	**24**	1.7	6.5	1.1	2.0	2.3	1.2	1.0	4.4	1.7	1.3	1.4	1.9	4.7	1.4	5.5	1.0	4.4	2.6	1.6
	**25**	1.3	2.4	2.7	2.0	3.1	1.7	1.9	1.5	1.7	1.0	3.7	2.1	1.3	1.0	1.9	1.8	1.1	2.6	1.6
	**26**	2.0	1.0	1.9	2.8	1.4	1.4	3.5	1.0	1.4	2.1	1.1	1.9	1.0	2.1	1.3	1.3	1.3	2.1	1.0
	**27**	2.0	1.0	2.4	1.9	1.3	1.3	2.5	1.0	1.2	2.6	1.0	4.3	1.4	1.1	1.0	3.0	1.7	1.3	1.3
	**28**	2.9	1.4	1.4	2.6	1.1	1.4	1.6	1.3	1.0	1.6	1.4	1.7	1.3	1.9	1.0	1.0	1.0	1.3	1.0
	**29**	3.0	1.3	2.1	1.4	2.5	2.6	1.1	2.4	4.2	1.9	1.4	3.0	1.6	1.0	2.3	1.3	1.6	1.3	2.1
	**30**	4.2	1.2	2.9	2.6	1.0	3.3	2.1	1.0	1.1	1.6	1.9	2.4	1.3	2.6	1.3	2.0	1.2	1.5	2.0
	**31**	1.0	3.7	1.6	1.0	4.0	1.0	1.3	3.6	4.0	1.1	2.3	2.5	5.0	1.1	3.3	1.2	2.1	1.4	1.4
	**32**	1.0	2.7	1.0	1.1	1.7	2.5	1.0	2.5	4.3	1.1	2.8	1.7	3.3	1.3	2.1	1.0	2.6	2.3	1.0
	**33**	1.4	2.4	1.1	1.0	1.9	2.2	1.7	2.0	3.9	1.4	3.1	1.8	2.6	1.1	2.3	1.3	1.6	1.0	1.0
	**34**	1.9	1.3	1.9	1.9	1.0	1.6	1.7	1.0	1.4	1.9	1.3	1.3	1.1	1.6	1.3	1.0	1.1	1.0	2.1
	**35**	1.3	1.0	2.1	3.2	1.0	1.4	2.7	1.5	1.3	1.0	1.6	1.0	1.0	1.1	1.3	3.3	1.3	1.4	1.7
	**36**	4.4	1.1	3.4	2.7	1.3	1.5	2.4	1.0	1.2	2.4	1.3	2.1	1.1	1.3	1.4	2.3	1.0	1.1	1.4
	**37**	1.1	1.7	1.9	1.9	1.4	2.0	1.9	1.6	1.7	2.1	1.4	2.1	1.3	1.0	1.6	2.0	1.1	3.0	1.1
	**38**	1.6	2.2	2.1	2.4	1.1	1.0	1.0	1.1	1.7	1.9	1.7	1.9	1.2	2.3	1.0	1.6	1.0	1.1	1.3
	**39**	2.7	1.3	3.1	1.9	1.1	3.3	2.7	1.0	1.1	2.3	1.6	2.4	1.0	1.0	1.1	2.0	1.0	1.9	1.3
	**40**	1.2	2.3	1.5	1.1	2.1	1.0	1.0	3.0	3.1	1.3	1.0	2.3	1.6	1.1	1.1	1.0	2.6	2.1	2.1
	**41**	3.0	1.6	3.5	4.2	1.0	1.6	2.1	1.0	1.1	3.2	1.4	2.9	1.1	4.1	1.6	1.8	1.0	1.6	1.3
	**42**	1.0	3.8	1.0	1.0	1.6	1.0	1.0	4.6	2.2	1.0	2.0	1.0	3.9	1.0	2.7	2.0	3.6	1.0	2.0

Note. Scenario pairs were rated on a 7-point scale from 1 = very different to 7 = very similar. Average ratings of 5.0 and higher are highlighted in blue.

Supplementary Table 3

Average similarity ratings for all combinations between original STEM scenarios and ChatGPT-created STEM scenarios; part 1 (original items 1-22)

		ChatGPT-created items																	
		**1**	**2**	**3**	**4**	**5**	**6**	**7**	**8**	**9**	**10**	**11**	**12**	**13**	**14**	**15**	**16**	**17**	**18**
**Original test items**	**1**	1.4	1.4	2.1	1.1	1.0	2.0	1.1	1.9	1.0	1.9	1.6	1.5	2.1	1.0	2.0	2.3	1.7	1.9
	**2**	4.2	1.3	1.1	1.0	3.1	1.0	1.0	2.0	1.1	1.3	1.0	5.0	1.1	1.0	1.6	1.1	1.9	1.6
	**3**	1.6	1.4	1.3	1.4	1.3	2.9	1.1	2.1	1.0	2.4	1.4	1.0	3.1	1.4	2.2	2.3	1.0	1.9
	**4**	1.7	1.7	1.9	1.0	1.0	1.9	1.0	1.4	1.0	1.6	1.0	1.0	1.7	1.1	1.3	1.8	1.1	2.3
	**5**	6.4	1.1	1.6	1.1	5.0	1.3	1.3	1.4	1.0	1.0	1.3	3.7	1.3	1.1	1.0	2.0	2.4	1.6
	**6**	2.1	1.1	4.3	1.1	1.3	1.0	2.1	1.7	1.0	3.6	1.0	1.1	1.1	5.6	2.3	1.3	1.0	2.1
	**7**	2.0	1.3	1.3	1.0	3.6	1.0	1.0	3.8	1.3	1.1	1.0	1.7	1.9	1.8	2.3	1.7	1.9	1.6
	**8**	1.9	2.4	1.9	1.3	1.0	2.7	1.3	1.3	1.0	1.1	1.7	1.1	2.3	1.0	2.1	1.6	1.1	3.3
	**9**	3.7	2.7	2.3	1.4	1.1	2.0	1.0	1.0	1.0	2.7	1.0	3.7	1.1	1.7	1.4	1.1	1.1	2.7
	**10**	1.1	1.4	5.9	1.0	1.1	2.0	1.7	1.9	1.0	4.7	1.0	1.6	2.1	2.7	1.7	2.4	1.1	1.3
	**11**	4.3	2.6	1.4	1.1	2.7	1.6	1.0	2.0	1.5	1.0	1.3	4.4	1.3	1.4	1.0	1.6	2.9	1.5
	**12**	1.0	1.2	2.6	2.0	1.6	3.1	1.0	2.6	1.3	1.5	1.7	1.0	2.1	1.1	2.5	3.4	1.1	2.4
	**13**	4.1	1.6	1.0	1.1	2.0	1.3	1.4	1.1	1.0	1.0	1.0	4.1	1.6	1.3	1.3	1.2	1.4	1.3
	**14**	1.6	2.1	1.9	1.4	1.3	1.1	1.0	1.4	2.2	1.3	2.0	1.1	1.1	1.1	1.9	1.6	1.9	1.7
	**15**	1.6	1.4	1.7	2.3	1.6	1.1	1.6	1.3	2.1	1.0	1.4	2.0	1.0	1.9	1.8	1.0	2.0	1.7
	**16**	1.6	1.3	1.9	1.7	1.0	4.2	1.1	2.3	1.1	1.7	3.0	1.0	4.0	1.0	1.7	2.6	1.7	3.0
	**17**	3.3	1.0	1.6	1.0	1.9	1.3	1.0	2.6	1.0	1.7	1.0	2.5	1.3	2.8	2.0	1.2	1.0	1.3
	**18**	1.7	2.3	1.0	1.5	1.3	1.6	1.0	2.3	3.0	1.7	1.1	1.0	1.8	1.0	1.4	2.1	1.1	2.9
	**19**	1.4	1.3	1.1	1.0	1.7	1.3	4.0	1.0	1.0	1.1	1.1	1.1	1.2	1.4	1.1	1.0	1.3	1.1
	**20**	1.9	1.4	1.0	2.4	1.3	2.0	1.3	1.8	1.6	1.6	1.9	1.1	2.3	1.0	1.0	1.0	2.4	2.0
	**21**	2.7	1.4	1.9	1.3	1.6	1.4	1.0	1.6	1.3	1.0	1.0	2.4	1.0	1.0	1.1	1.5	1.6	1.4
	**22**	1.0	1.1	1.1	3.7	1.0	1.0	1.0	1.1	4.0	1.1	5.7	1.6	1.7	1.0	1.3	1.2	1.0	1.7

Note. Scenario pairs were rated on a 7-point scale from 1 = very different to 7 = very similar. Average ratings of 5.0 and higher are highlighted in blue.

Supplementary Table 4

Average similarity ratings for all combinations between original STEM scenarios and ChatGPT-created STEM scenarios; part 2 (original items 23-44)

	ChatGPT-created items																	
**Original test items**	**1**	**2**	**3**	**4**	**5**	**6**	**7**	**8**	**9**	**10**	**11**	**12**	**13**	**14**	**15**	**16**	**17**	**18**
**23**	1.8	2.0	3.6	1.0	1.1	1.3	1.9	2.8	1.0	2.3	1.1	2.0	1.6	2.3	1.4	2.4	1.0	1.6
**24**	1.0	1.4	1.4	1.1	1.1	1.3	1.4	1.4	3.0	1.5	4.3	1.3	1.3	1.6	1.6	1.3	1.3	3.3
**25**	3.1	2.0	1.9	1.1	1.1	1.3	1.0	1.9	1.6	1.1	1.1	3.9	1.0	1.1	1.6	1.0	2.1	1.3
**26**	2.3	4.6	1.4	1.3	1.1	1.0	1.1	2.1	2.0	1.4	1.0	1.6	1.4	1.0	1.0	1.7	2.9	2.3
**27**	1.9	1.1	2.7	1.3	1.4	3.2	1.3	3.5	1.3	2.5	1.0	1.3	2.1	1.0	2.7	3.0	1.1	2.0
**28**	2.8	1.4	1.3	1.0	2.4	1.0	3.0	1.0	1.4	1.0	1.0	2.2	1.3	1.7	1.0	1.0	1.6	1.0
**29**	1.6	1.8	1.3	1.7	1.6	1.3	1.0	1.4	1.7	1.0	2.6	1.0	1.3	1.1	1.7	1.1	1.9	1.7
**30**	1.3	1.4	5.3	1.2	1.1	1.1	1.1	1.9	1.0	5.7	1.1	1.0	1.6	3.1	1.3	1.1	1.0	1.3
**31**	1.7	1.3	2.7	1.0	2.4	2.1	2.9	2.6	2.1	1.6	1.0	1.4	2.1	1.1	2.1	2.5	1.0	1.6
**32**	5.7	1.3	1.0	1.3	3.0	1.4	1.0	2.3	1.0	1.3	1.0	3.8	1.0	1.1	1.7	1.6	1.0	1.9
**33**	2.1	3.2	3.3	1.6	2.0	1.9	1.1	2.6	1.6	1.5	2.3	1.4	2.9	1.4	2.1	1.3	1.0	2.1
**34**	5.3	1.3	1.9	1.0	4.8	1.7	1.0	1.3	1.1	1.1	1.0	3.6	1.9	1.3	1.6	1.9	1.7	1.0
**35**	1.1	3.0	2.1	1.3	1.8	2.1	1.2	2.0	1.3	1.0	1.0	1.3	1.9	1.4	2.1	1.0	1.3	1.3
**36**	5.0	1.6	1.0	1.1	3.9	1.0	1.1	1.3	1.1	1.0	1.0	3.4	1.1	1.6	1.9	1.5	1.7	1.1
**37**	1.6	1.7	3.0	1.0	1.7	1.3	4.4	1.0	1.3	2.0	1.0	1.6	1.0	3.8	1.4	1.3	1.1	1.7
**38**	1.6	1.7	1.3	1.1	1.1	1.8	1.1	1.9	1.0	1.9	1.1	1.6	2.2	1.0	1.6	2.3	1.0	1.7
**39**	1.6	1.4	1.5	2.2	1.3	2.6	1.1	1.1	1.1	1.1	1.6	1.6	2.4	1.0	2.4	1.1	1.0	2.1
**40**	2.0	1.0	2.6	1.0	2.0	1.7	1.6	3.3	1.8	1.4	1.0	1.0	2.6	1.0	1.6	2.0	1.3	1.3
**41**	1.0	1.1	1.0	1.1	1.0	1.0	1.3	1.1	1.7	1.0	1.6	1.1	1.0	1.5	1.0	1.3	1.3	1.3
**42**	1.8	1.8	2.4	2.3	1.1	1.4	1.0	2.1	2.5	1.0	1.0	1.3	1.9	1.3	2.6	2.3	1.0	1.7
**43**	1.9	1.1	3.6	1.0	1.0	1.0	2.4	1.5	1.6	1.9	1.4	2.3	1.1	3.1	1.0	1.1	1.7	1.5
**44**	2.7	2.1	1.4	1.0	3.6	1.7	1.0	1.3	1.1	1.0	1.4	2.0	1.3	1.9	1.3	1.0	2.3	1.5

Note. Scenario pairs were rated on a 7-point scale from 1 = very different to 7 = very similar. Average ratings of 5.0 and higher are highlighted in blue.

Supplementary Table 5

Average similarity ratings for all combinations between original GEMOK scenarios and ChatGPT-created GEMOK scenarios

	ChatGPT-created items																			
**Original test items**	**1**	**2**	**3**	**4**	**5**	**6**	**7**	**8**	**9**	**10**	**11**	**12**	**13**	**14**	**15**	**16**	**17**	**18**	**19**	**20**
**1**	2.4	2.1	1.0	1.8	1.4	1.6	1.3	2.0	1.4	1.6	1.8	1.6	1.4	1.0	2.6	2.0	1.9	2.7	1.7	1.1
**2**	2.9	5.3	1.7	1.0	2.1	1.9	2.8	1.6	2.2	2.5	1.1	1.7	4.5	1.3	1.2	1.9	2.5	2.3	2.0	3.6
**3**	2.2	2.0	1.3	1.7	2.4	1.3	1.5	1.6	2.6	1.4	2.0	2.0	1.3	2.3	1.5	1.0	2.1	1.6	1.2	1.5
**4**	1.8	1.5	1.5	2.0	1.4	1.6	1.8	1.9	1.7	1.9	1.8	2.2	1.1	2.3	2.3	1.4	2.2	1.0	1.0	1.5
**5**	1.4	1.6	2.1	2.7	1.8	1.7	1.8	1.2	1.6	1.7	1.4	1.6	1.5	1.3	2.0	2.1	1.3	1.9	1.0	1.6
**6**	1.4	1.6	1.3	1.5	1.2	1.3	1.7	2.2	2.8	1.3	1.4	1.3	1.1	1.5	2.0	1.9	1.3	1.3	1.8	1.3
**7**	1.5	3.1	1.7	1.5	2.0	3.3	1.8	2.0	1.5	2.0	1.9	1.5	1.6	1.6	1.8	2.3	2.0	1.5	1.7	1.3
**8**	1.0	1.8	2.0	2.1	1.0	1.3	1.9	1.4	1.7	1.1	1.6	1.7	1.8	1.0	1.5	1.1	1.0	1.6	1.1	1.4
**9**	3.0	2.2	2.2	1.8	1.9	2.1	1.0	1.7	2.0	1.8	2.0	2.1	1.6	1.1	2.0	1.3	3.0	1.6	2.0	2.0
**10**	1.7	1.7	1.8	1.4	2.0	1.7	1.5	1.7	1.9	1.1	1.3	2.0	1.3	1.7	3.9	3.4	1.7	1.0	2.0	1.1
**11**	1.2	1.4	1.3	1.7	1.3	1.1	1.7	1.4	1.3	1.1	1.4	1.1	1.1	1.9	1.9	3.6	1.1	1.4	1.0	1.1
**12**	2.7	4.0	1.8	1.4	2.1	1.3	2.3	2.1	1.3	1.4	2.0	2.6	1.5	1.7	1.3	1.6	1.8	2.3	1.2	2.1
**13**	1.0	1.3	2.0	1.8	1.7	1.3	2.0	1.9	1.6	1.9	2.7	2.8	1.0	1.7	1.5	1.6	1.1	1.0	1.3	1.2
**14**	1.3	1.3	1.4	1.3	1.0	1.3	1.1	1.4	2.0	1.8	1.1	1.3	1.6	1.5	1.5	1.3	1.3	1.8	1.7	2.0
**15**	2.0	2.6	2.4	1.3	2.1	1.4	1.6	1.3	1.2	1.4	1.3	1.3	1.9	1.3	1.3	1.8	1.7	1.9	2.9	1.3
**16**	1.3	1.8	1.2	1.3	1.7	1.1	1.4	1.1	1.5	1.7	1.9	1.1	1.0	2.0	2.3	1.8	1.3	1.0	1.8	1.3
**17**	1.4	1.7	1.4	2.3	1.4	2.5	1.5	1.4	2.0	1.2	1.0	2.7	1.8	1.3	1.3	2.3	1.7	1.8	1.3	1.7
**18**	1.3	1.5	3.2	1.4	2.9	2.0	2.3	1.5	2.0	1.1	3.2	1.2	2.7	1.3	1.3	1.8	2.0	1.7	2.0	1.7
**19**	2.1	1.5	1.5	2.0	1.2	1.7	1.0	1.2	1.6	2.0	1.5	1.7	2.0	1.3	1.0	2.3	1.3	1.0	1.3	1.2
**20**	1.5	2.0	2.0	2.8	2.3	1.9	1.3	2.5	2.3	1.8	1.0	1.6	2.0	1.1	2.3	1.6	2.3	1.7	1.8	1.4

Note. Scenario pairs were rated on a 7-point scale from 1 = very different to 7 = very similar. Average ratings of 5.0 and higher are highlighted in blue.

Supplementary Table 6

Average similarity ratings for all combinations between original GECo Regulation scenarios and ChatGPT-created GECo Regulation scenarios; part 1 (ChatGPT-created scenarios 1-18)

	ChatGPT-created items																	
**Original test items**	**1**	**2**	**3**	**4**	**5**	**6**	**7**	**8**	**9**	**10**	**11**	**12**	**13**	**14**	**15**	**16**	**17**	**18**
**1**	1.9	1.5	2.0	1.4	1.0	1.7	1.1	1.3	1.4	2.3	1.6	1.3	2.4	1.9	1.5	1.2	1.5	1.1
**2**	2.0	2.0	1.9	3.0	3.3	2.5	5.0	2.3	2.0	3.7	2.7	3.0	2.0	1.7	3.0	2.2	3.3	2.3
**3**	2.7	5.2	1.7	1.4	1.9	2.0	2.0	2.1	1.1	2.8	2.5	2.3	1.5	1.9	4.3	1.0	1.9	1.6
**4**	3.3	2.1	2.0	1.8	3.3	1.5	2.9	1.4	2.1	1.5	3.7	2.8	1.7	2.0	1.8	3.1	3.3	3.0
**5**	1.0	1.6	1.4	1.3	1.0	1.6	2.0	1.3	1.3	2.7	1.6	3.5	1.5	1.7	1.0	1.8	2.0	1.6
**6**	1.8	2.0	1.4	2.3	1.6	1.3	1.6	1.9	1.9	2.0	1.3	1.3	2.7	2.0	2.0	2.0	2.6	2.0
**7**	3.0	2.1	2.6	1.1	3.5	1.7	2.3	2.4	3.3	1.4	5.1	3.6	1.0	1.9	2.4	5.6	2.9	2.9
**8**	1.4	1.3	1.1	1.9	2.0	1.4	1.0	2.8	1.4	1.6	1.3	1.1	1.0	1.4	1.0	1.4	1.7	1.0
**9**	1.7	3.2	3.6	1.5	1.6	2.7	1.4	3.2	3.0	1.9	2.7	3.4	1.0	1.8	1.8	2.0	1.4	2.2
**10**	2.1	4.0	1.9	1.7	1.3	1.6	1.7	2.9	6.1	3.1	2.7	2.2	1.9	1.8	2.7	1.5	1.7	1.4
**11**	1.4	2.1	1.1	1.4	1.4	1.7	1.9	1.3	1.8	2.1	2.5	1.4	1.0	1.7	1.5	2.0	1.3	1.0
**12**	2.5	5.7	1.7	2.7	1.4	1.8	3.3	3.7	1.7	2.9	1.3	2.1	1.8	1.7	3.7	2.8	1.7	2.3
**13**	1.0	1.7	5.0	1.3	1.7	3.8	1.3	2.3	3.8	1.3	1.2	3.6	1.6	1.9	2.2	1.6	1.1	2.1
**14**	1.4	4.0	1.4	2.0	2.7	2.5	3.7	2.9	1.6	1.3	3.0	2.8	1.7	2.7	3.0	1.5	1.9	3.0
**15**	2.7	2.0	1.7	1.1	1.0	1.5	2.3	1.5	1.0	2.3	1.6	2.0	1.8	2.0	1.6	1.3	1.9	2.2
**16**	1.7	3.1	1.7	1.3	3.0	2.3	2.8	1.6	1.7	1.5	1.7	2.4	2.3	1.8	2.3	1.6	1.5	1.7
**17**	1.6	2.2	1.0	1.3	1.0	1.3	1.7	1.6	1.7	1.2	2.0	1.4	1.4	1.7	1.4	1.5	1.4	1.3
**18**	1.4	1.3	1.4	1.1	1.2	1.7	1.0	2.0	1.7	1.6	1.0	1.0	1.4	1.7	3.3	1.3	1.3	1.0
**19**	1.5	1.3	2.0	1.3	1.4	2.0	1.4	1.5	1.7	1.3	2.2	1.3	1.5	2.0	1.1	1.6	1.6	1.0
**20**	3.7	1.1	2.6	1.3	4.7	1.8	1.9	1.6	1.4	1.6	3.4	1.5	1.4	1.7	1.2	2.4	2.7	1.5
**21**	1.0	1.3	2.3	1.3	2.0	1.1	1.3	2.3	2.1	1.1	1.8	1.4	1.1	1.4	1.3	2.1	2.4	2.0
**22**	1.1	2.0	1.3	1.1	1.0	1.4	1.0	1.4	1.0	1.0	1.0	1.3	1.2	1.0	1.3	1.0	1.4	1.5
**23**	2.0	1.5	2.0	1.2	1.9	2.0	2.1	1.4	1.3	2.7	3.2	1.7	1.0	1.0	1.0	1.0	4.7	1.8
**24**	3.4	1.1	2.0	1.3	3.2	1.7	2.0	1.6	1.9	2.1	3.0	2.1	1.8	2.4	1.5	1.5	3.9	2.8
**25**	1.7	3.2	1.2	1.1	1.0	2.1	1.0	1.3	1.0	1.1	1.3	1.0	1.2	1.0	1.0	2.0	1.0	1.3
**26**	1.1	2.1	1.7	3.6	1.6	1.4	1.0	1.5	1.9	2.0	2.3	1.6	1.3	1.1	1.1	1.2	1.7	2.4
**27**	4.0	2.0	3.2	1.8	2.8	2.1	2.4	2.5	3.2	1.9	4.1	1.9	1.3	4.7	1.7	2.6	2.1	4.6
**28**	1.4	2.3	1.5	1.3	1.6	1.8	1.4	1.4	1.7	2.6	1.3	2.3	1.0	1.4	1.3	3.3	2.8	1.5

Supplementary Table 7

Average similarity ratings for all combinations between original GECo Regulation scenarios and ChatGPT-created GECo Regulation scenarios; part 2 (ChatGPT-created scenarios 19-28)

		ChatGPT-created items									
		**19**	**20**	**21**	**22**	**23**	**24**	**25**	**26**	**27**	**28**
**Original test items**	**1**	1.2	2.1	1.0	1.4	1.6	1.7	2.0	1.1	1.6	1.1
	**2**	1.7	2.1	2.0	2.9	2.1	2.8	2.3	3.2	1.8	4.0
	**3**	2.4	1.0	2.3	1.4	1.7	2.0	1.3	1.6	1.4	2.0
	**4**	2.0	3.3	2.2	2.4	2.4	1.3	1.8	4.0	2.2	2.0
	**5**	2.6	1.0	1.3	2.3	3.6	2.4	1.0	1.6	1.3	1.7
	**6**	1.4	1.9	2.2	1.3	1.6	1.4	1.6	1.1	1.3	1.1
	**7**	1.7	3.1	4.3	3.0	1.6	3.1	2.5	3.0	3.9	2.7
	**8**	2.3	2.4	1.4	1.2	1.3	1.3	1.0	1.5	1.1	1.7
	**9**	1.6	2.0	2.3	1.7	1.3	1.4	1.4	1.3	3.2	2.0
	**10**	1.7	1.0	1.4	2.2	1.1	1.6	1.9	2.0	2.2	2.2
	**11**	1.1	2.7	1.8	1.1	1.1	2.2	1.8	1.0	1.6	2.0
	**12**	2.2	1.9	4.4	2.1	1.5	2.2	1.8	1.3	2.4	2.7
	**13**	1.1	1.2	1.4	2.7	1.4	1.8	1.7	1.4	4.1	1.1
	**14**	2.1	1.6	2.4	2.3	2.0	2.6	3.8	1.0	1.1	2.2
	**15**	1.1	1.7	1.0	1.6	3.4	1.6	1.1	1.2	1.3	1.8
	**16**	1.9	1.4	4.2	1.4	1.3	1.4	1.6	2.6	3.0	2.4
	**17**	1.1	2.9	1.9	1.8	1.3	1.3	1.2	1.0	1.3	1.0
	**18**	1.5	1.6	1.3	1.5	1.3	1.3	1.6	1.1	2.0	1.5
	**19**	1.3	2.4	1.7	2.6	1.0	1.0	1.8	1.7	2.3	1.4
	**20**	1.9	2.7	1.7	3.0	3.0	3.6	1.9	4.3	1.3	2.8
	**21**	1.0	1.9	1.5	2.4	1.3	1.9	1.4	1.8	1.0	1.6
	**22**	1.0	1.1	1.4	1.8	1.0	1.1	1.1	1.0	1.0	1.0
	**23**	2.1	2.4	1.4	1.9	6.7	3.0	2.0	3.8	1.2	2.4
	**24**	2.0	1.9	1.1	3.5	1.6	3.9	1.7	4.8	1.7	4.3
	**25**	1.0	1.0	1.0	1.0	1.0	1.0	1.7	1.1	1.7	1.1
	**26**	4.0	1.1	2.3	1.4	2.3	3.4	1.6	2.0	1.2	1.7
	**27**	4.0	1.6	2.7	3.8	1.7	4.2	1.9	2.6	2.1	3.3
	**28**	1.0	2.1	1.6	1.3	3.0	1.3	1.4	1.1	1.6	1.7

Supplementary Table 8

Average similarity ratings for all combinations between original GECo Management scenarios and ChatGPT-created GECo Management scenarios

		ChatGPT-created items																			
		**1**	**2**	**3**	**4**	**5**	**6**	**7**	**8**	**9**	**10**	**11**	**12**	**13**	**14**	**15**	**16**	**17**	**18**	**19**	**20**
**Original test items**	**1**	1.5	4.0	1.8	2.3	1.3	1.8	1.9	1.7	1.3	1.0	1.3	1.8	1.3	1.3	2.0	1.3	1.8	1.5	1.6	2.6
	**2**	2.7	3.4	1.2	4.0	1.1	2.0	2.0	1.2	1.1	1.6	1.3	1.3	3.0	2.4	1.4	2.0	1.7	1.7	1.3	2.7
	**3**	2.0	3.1	2.9	2.6	2.4	3.6	2.3	1.3	1.0	1.8	1.6	2.3	1.7	2.7	4.8	1.8	1.3	2.0	2.0	3.3
	**4**	2.4	1.5	1.7	3.3	2.5	1.2	1.6	2.7	1.0	3.2	2.5	2.0	1.5	3.2	2.7	1.5	1.8	2.5	1.5	2.5
	**5**	1.7	2.0	1.8	2.3	1.3	2.0	1.7	2.7	1.2	1.7	1.4	1.7	2.3	1.7	1.9	1.7	1.3	1.8	1.7	2.7
	**6**	2.5	1.9	2.0	3.0	2.6	3.2	1.3	2.7	1.4	2.0	1.6	1.3	2.0	1.8	1.1	1.0	2.0	3.0	1.7	1.7
	**7**	1.8	2.2	2.0	2.0	1.1	1.5	1.3	1.1	1.5	1.6	2.0	1.2	3.1	2.9	1.5	1.3	1.3	1.3	2.7	2.3
	**8**	2.3	1.6	1.0	1.5	1.5	1.4	1.7	1.9	1.3	1.3	3.2	2.3	3.0	3.6	1.0	1.4	1.5	1.7	1.3	1.5
	**9**	2.0	2.2	1.6	2.5	1.5	2.8	1.8	1.6	1.2	2.2	1.7	2.0	2.3	1.7	3.0	1.7	1.1	1.8	1.0	1.8
	**10**	4.3	1.8	1.3	2.2	1.3	1.4	1.8	1.3	1.1	1.7	3.5	1.7	2.4	3.0	1.7	2.0	1.7	3.3	1.8	1.2
	**11**	1.9	4.7	2.0	2.1	2.1	2.0	1.6	1.3	1.3	1.4	1.1	1.4	2.3	1.6	2.6	2.0	1.2	2.0	1.5	1.5
	**12**	1.3	1.2	1.2	1.2	1.3	1.3	1.2	1.5	1.4	1.0	2.0	1.0	1.3	1.0	2.0	1.1	1.5	1.7	2.0	1.1
	**13**	2.0	1.3	1.1	2.2	1.9	1.7	1.6	1.3	1.7	1.2	1.3	1.6	1.8	1.7	1.2	1.1	2.2	1.5	1.1	1.7
	**14**	1.4	1.5	2.0	1.5	1.7	1.5	1.3	1.8	1.6	1.7	1.7	1.3	1.5	1.8	1.3	1.2	1.5	1.0	1.0	2.1
	**15**	4.0	3.8	3.2	2.5	1.7	2.5	3.8	2.2	1.3	2.8	1.5	1.9	3.0	3.6	2.8	1.2	1.8	1.7	2.4	2.3
	**16**	1.5	1.3	1.7	1.5	1.0	2.0	1.2	1.2	1.0	1.7	2.7	1.8	2.0	1.5	1.5	1.7	1.3	1.3	1.1	1.4
	**17**	1.5	1.3	1.3	1.2	1.8	1.8	1.6	2.0	1.8	1.0	3.0	1.3	1.7	1.3	1.0	2.0	1.1	1.7	1.3	1.8
	**18**	1.7	1.3	1.1	1.2	1.0	1.0	1.7	2.2	1.0	1.0	2.7	2.0	2.5	1.7	3.0	1.5	2.3	1.0	1.3	2.4
	**19**	1.5	1.2	1.1	1.3	1.6	1.5	1.3	1.3	1.2	1.2	2.7	1.3	1.5	1.6	1.5	1.2	1.4	1.3	2.2	1.8
	**20**	2.9	3.0	2.0	2.3	1.0	1.8	2.0	2.3	1.1	1.5	2.7	1.0	3.8	2.7	1.1	2.5	1.5	1.7	1.2	2.5

Note. Scenario pairs were rated on a 7-point scale from 1 = very different to 7 = very similar. Average ratings of 5.0 and higher are highlighted in blue.

Supplementary Notes 4: Item-level characteristics for ChatGPT-generated and original tests

The following Tables include item-level mean scores, item-total correlations, and clarity and realism ratings for both test versions, enabling readers to compare the original and ChatGPT-generated test versions and explore potential reasons for differences.

As an example, we conducted a more detailed analysis of the STEU-B, as this was the only test where the ChatGPT-generated version exhibited a significantly lower Cronbach's alpha compared to the original version.

At the test-level (as detailed in Tables 6–9 of the main manuscript), there was no apparent reason for the lower alpha in the ChatGPT-generated version, as there was no substantial difference in mean difficulty, content diversity, and correlations with other tests. Clarity and realism ratings were even higher in the ChatGPT-generated version. However, at the item level, four items (4, 7, 8, and 13) showed negative item-total correlations of .10 or higher, contributing to the low alpha (see Table S9a). Among these, three items (7, 8, and 13) had low mean scores (.48, .10, .30), and for one additional item (18), no participant selected the response deemed correct by ChatGPT.

Examining the item texts and response options revealed potential issues. For items 7, 8, and 13, the five response options were relatively similar within each item. For example, in item 13 (about a runner crossing the finish line), the options included excitement, joy, pride, relief, and surprise, where multiple options (or a blend of emotions) could theoretically be considered correct without additional contextual details in the item text. If further test development is pursued, these items could be improved by providing more distinct response options, adding more detailed cues to the scenarios, replacing the items entirely, or adopting consensus scoring (as used in the original STEU) by designating the most frequently chosen option as correct.

To illustrate the potential impact of this last approach, we recoded items 7, 8, 13, and 18 to mark the most frequently chosen option as correct. For item 7, a secondary option was almost as frequently selected (mean scores: .47 versus .48) and was also recoded as correct. Following these adjustments, the item-total correlations for items 7, 13, and 18 became positive, raising Cronbach's alpha to .35. While still low by conventional standards, this value approaches the .41 of the original STEU-B. However, item 8 continued to exhibit a negative item-total correlation, indicating that it would likely be replaced in future test versions.

To put the presence of certain poorly performing items in the ChatGPT-generated test versions into perspective, it is important to emphasize that the goal of this project was not to develop finalized test versions for assessing ability EI. Rather, the aim was to determine whether ChatGPT could, in principle, generate appropriate item sets without further modifications. Specifically, our focus was on assessing the psychometric quality of the full tests as an indicator of ChatGPT's emotion knowledge, rather than evaluating individual items. The presence of poorly performing items in the newly generated test versions should be interpreted in light of the highly stringent nature of our comparison. We examined the psychometric quality of ChatGPT's initial item pool and compared it to published tests, which had undergone multiple construction phases, starting with larger item pools that were iteratively revised and refined.

Supplementary Table 9a

Item characteristics of ChatGPT-generated items for STEU-B.

Item number	mean score	clarity	realism	item-total correlation	item-total correlation [a]
STEU_gpt_item1	.81	83.3	87.7	.08	.07
STEU_gpt_item2	.88	93.6	94.1	.01	-.01
STEU_gpt_item3	.44	85.8	90.4	.03	.04
STEU_gpt_item4	.78	93.2	94.4	-.14	-.06
STEU_gpt_item5	.81	90.5	88.7	.13	.21
STEU_gpt_item6	.77	90.7	87.9	.06	.06
STEU_gpt_item7	.48	89.6	90.3	-.18	.17
STEU_gpt_item8	.10	88.6	89.4	-.13	-.16
STEU_gpt_item9	.97	78.7	83.8	.30	.31
STEU_gpt_item10	.23	83.1	84.7	-.01	.04
STEU_gpt_item11	.99	90.0	86.7	.22	.22
STEU_gpt_item12	.76	69.9	76.0	.23	.37
STEU_gpt_item13	.30	91.0	91.7	-.15	.14
STEU_gpt_item14	.80	65.9	66.9	.25	.22
STEU_gpt_item15	.46	86.2	88.2	.10	.08
STEU_gpt_item16	.64	87.3	88.8	.12	.26
STEU_gpt_item17	.71	80.5	88.4	-.08	-.05
STEU_gpt_item18	.00	88.4	84.0	.00	.16
STEU_gpt_item19	.91	75.9	80.6	.07	.14

Note: [a] item-total correlations after recoding items 7, 8, 13, and 18 (see text at beginning of section IV).

Supplementary Table 9b

Item characteristics of original items for STEU-B.

Item number	mean score	clarity	realism	item-total correlation
STEU_original_item1	.47	87.4	87.7	.15
STEU_original_item2	.62	83.6	94.1	.11
STEU_original_item3	.33	81.9	90.4	.19
STEU_original_item4	.24	86.7	94.4	-.05
STEU_original_item5	.19	63.7	88.7	.11
STEU_original_item6	.95	84.5	87.9	.12
STEU_original_item7	.77	74.0	90.3	.34
STEU_original_item8	.89	70.7	89.4	.09
STEU_original_item9	.83	69.9	83.8	.09
STEU_original_item10	.80	81.7	84.7	.21
STEU_original_item11	.87	69.2	86.7	.13
STEU_original_item12	.81	83.8	76.0	.17
STEU_original_item13	.62	79.3	91.7	.13
STEU_original_item14	.73	65.7	66.9	.30
STEU_original_item15	.70	81.2	88.2	.25
STEU_original_item16	.56	80.9	88.8	-.05
STEU_original_item17	.80	73.3	88.4	-.06
STEU_original_item18	.31	59.5	84.0	-.03
STEU_original_item19	.60	75.0	80.6	.13

Supplementary Table 10a

Item characteristics of ChatGPT-generated items for STEM-B.

Item number	mean score	clarity	realism	item-total correlation
STEM_gpt_item1	.97	92.4	90.9	.41
STEM_gpt_item2	.91	83.7	82.2	.37
STEM_gpt_item3	.99	93.4	91.4	.07
STEM_gpt_item4	.98	91.1	88.0	.10
STEM_gpt_item5	.99	90.5	93.2	.44
STEM_gpt_item6	.98	86.3	85.5	.04
STEM_gpt_item7	.99	92.5	92.7	.44
STEM_gpt_item8	.97	89.7	88.7	.13
STEM_gpt_item9	1.00	90.2	86.7	.00
STEM_gpt_item10	.87	90.6	89.4	.25
STEM_gpt_item11	.97	91.3	89.0	.30
STEM_gpt_item12	1.00	91.2	88.6	.00
STEM_gpt_item13	.96	86.8	83.9	.35
STEM_gpt_item14	.99	90.3	89.2	.25
STEM_gpt_item15	1.00	86.7	81.1	.00
STEM_gpt_item16	.98	87.9	85.2	.24
STEM_gpt_item17	.99	88.2	81.0	.44
STEM_gpt_item18	.29	80.6	81.0	.18

Supplementary Table 10b

Item characteristics of original items for STEM-B.

Item number	mean score	clarity	realism	item-total correlation
STEM_original_item1	.61	90.8	91.4	.14
STEM_original_item2	.62	85.6	87.2	.01
STEM_original_item3	.67	85.1	86.5	.10
STEM_original_item4	.71	88.1	88.8	.33
STEM_original_item5	.76	85.1	86.5	.17
STEM_original_item6	.82	85.7	86.7	.19
STEM_original_item7	.83	86.2	88.9	.34
STEM_original_item8	.69	86.9	88.6	.23
STEM_original_item9	.56	80.4	82.4	.08
STEM_original_item10	.47	80.5	86.3	.20
STEM_original_item11	.79	86.2	86.4	.11
STEM_original_item12	.60	80.2	85.1	.25
STEM_original_item13	.89	83.7	82.6	.18
STEM_original_item14	.93	88.9	88.6	.28
STEM_original_item15	.52	82.0	85.5	-.04
STEM_original_item16	.44	83.3	83.3	.09
STEM_original_item17	.71	85.3	85.8	.15
STEM_original_item18	.88	82.4	83.1	.08

Supplementary Table 11a

Item characteristics of ChatGPT-generated items for GEMOK Blends.

Item number	mean score	clarity	realism	item-total correlation
GEMOK_gpt_item1	.43	79.2	83.7	.11
GEMOK_gpt_item2	.19	86.8	88.4	.09
GEMOK_gpt_item3	.63	80.1	81.5	.10
GEMOK_gpt_item4	.11	75.6	83.4	.18
GEMOK_gpt_item5	.51	67.6	72.4	.08
GEMOK_gpt_item6	.09	63.2	66.3	.06
GEMOK_gpt_item7	.91	80.1	82.4	.37
GEMOK_gpt_item8	.53	75.6	75.8	.07
GEMOK_gpt_item9	.15	69.3	67.7	-.11
GEMOK_gpt_item10	.47	65.8	75.9	.18
GEMOK_gpt_item11	.85	72.1	63.5	.35
GEMOK_gpt_item13	.42	64.8	77.9	.22
GEMOK_gpt_item14	.40	58.5	61.7	.00
GEMOK_gpt_item15	.68	80.4	84.1	.26
GEMOK_gpt_item16	.04	73.4	63.8	-.03
GEMOK_gpt_item17	.46	76.6	76.8	.13
GEMOK_gpt_item18	.96	82.7	83.6	.13
GEMOK_gpt_item19	.90	73.9	74.1	.32
GEMOK_gpt_item20	.16	72.2	74.5	.06

Note. Item 12 is missing because it was presented to participants with the wrong response options and was therefore excluded from analyses.

Supplementary Table 11b

Item characteristics of original items for GEMOK Blends.

Item number	mean score	clarity	realism	item-total correlation
GEMOK_original_item1	.76	88.9	90.3	.15
GEMOK_original_item2	.87	93.8	92.2	.20
GEMOK_original_item3	.54	88.6	84.4	.30
GEMOK_original_item4	.46	82.2	71.7	.20
GEMOK_original_item5	.21	85.7	85.9	-.01
GEMOK_original_item6	.74	84.4	83.1	.23
GEMOK_original_item7	.46	76.3	72.8	.10
GEMOK_original_item8	.68	79.8	74.2	.22
GEMOK_original_item9	.66	87.0	86.7	.26
GEMOK_original_item10	.73	88.1	85.3	.39
GEMOK_original_item11	.78	86.6	78.4	.07
GEMOK_original_item12	.58	92.7	92.3	-.01
GEMOK_original_item13	.85	88.0	86.6	.09
GEMOK_original_item14	.64	83.2	74.3	.07
GEMOK_original_item15	.82	90.2	78.0	.32
GEMOK_original_item16	.93	83.7	74.4	.21
GEMOK_original_item17	.82	83.9	75.2	.24
GEMOK_original_item18	.82	87.3	85.0	.26
GEMOK_original_item19	.75	84.6	80.8	.31
GEMOK_original_item20	.49	83.9	82.2	.22

Supplementary Table 12a

Item characteristics of ChatGPT-created items for GECO Emotion Regulation subtest.

Item number	mean score	clarity	realism	item-total correlation
GECo_ER_gpt_item1	.64	85.5	77.3	.39
GECo_ER_gpt_item2	.69	84.2	82.1	.52
GECo_ER_gpt_item3	.41	84.3	77.1	.41
GECo_ER_gpt_item4	.56	81.8	75.4	.45
GECo_ER_gpt_item5	.61	82.9	77.6	.48
GECo_ER_gpt_item6	.67	84.0	78.9	.50
GECo_ER_gpt_item7	.54	83.1	74.2	.61
GECo_ER_gpt_item8	.73	82.7	75.2	.53
GECo_ER_gpt_item9	.68	82.5	78.4	.64
GECo_ER_gpt_item10	.70	82.9	78.7	.50
GECo_ER_gpt_item11	.61	80.7	77.9	.67
GECo_ER_gpt_item12	.64	81.9	76.9	.71
GECo_ER_gpt_item13	.75	81.5	77.8	.65
GECo_ER_gpt_item14	.85	81.9	76.2	.60
GECo_ER_gpt_item15	.64	83.9	79.7	.69
GECo_ER_gpt_item16	.69	81.5	75.5	.63
GECo_ER_gpt_item17	.53	83.1	77.3	.65
GECo_ER_gpt_item18	.64	82.0	76.2	.67
GECo_ER_gpt_item19	.62	81.0	78.6	.64
GECo_ER_gpt_item20	.61	81.1	74.1	.65
GECo_ER_gpt_item21	.72	81.8	75.4	.54
GECo_ER_gpt_item22	.58	81.1	73.7	.67
GECo_ER_gpt_item23	.56	84.8	75.2	.52
GECo_ER_gpt_item24	.67	81.6	75.1	.55
GECo_ER_gpt_item25	.71	82.2	76.5	.61
GECo_ER_gpt_item26	.63	83.0	77.9	.63
GECo_ER_gpt_item27	.81	83.1	76.3	.52
GECo_ER_gpt_item28	.82	83.4	77.0	.62

Supplementary Table 12b

Item characteristics of original items for GECO Emotion Regulation subtest.

Item number	mean score	clarity	realism	item-total correlation
GECo_ER_original_item1	.28	75.3	69.2	-.02
GECo_ER_original_item2	.63	84.4	76.7	.27
GECo_ER_original_item3	.73	84.4	79.2	.10
GECo_ER_original_item4	.55	83.7	73.3	.32
GECo_ER_original_item5	.58	81.5	74.3	.37
GECo_ER_original_item6	.40	81.8	76.9	.54
GECo_ER_original_item7	.29	80.1	69.1	.47
GECo_ER_original_item8	.75	77.6	67.4	.23
GECo_ER_original_item9	.42	82.9	70.0	.32
GECo_ER_original_item10	.64	84.4	78.0	.48
GECo_ER_original_item11	.62	84.3	77.9	.38
GECo_ER_original_item12	.72	80.3	77.3	.43
GECo_ER_original_item13	.50	82.4	75.1	.42
GECo_ER_original_item14	.35	80.1	72.4	.03
GECo_ER_original_item15	.71	75.9	69.4	.31
GECo_ER_original_item16	.58	80.9	75.1	.35
GECo_ER_original_item17	.34	81.4	72.3	-.42
GECo_ER_original_item18	.56	85.1	78.3	.35
GECo_ER_original_item19	.52	78.0	71.9	.19
GECo_ER_original_item20	.66	83.5	77.6	.35
GECo_ER_original_item21	.43	82.6	75.8	.33
GECo_ER_original_item22	.57	77.7	69.7	.03
GECo_ER_original_item23	.61	83.3	78.2	.30
GECo_ER_original_item24	.64	77.7	70.7	.41
GECo_ER_original_item25	.51	76.4	69.5	.27
GECo_ER_original_item26	.53	79.1	70.6	.34
GECo_ER_original_item27	.55	80.1	72.0	.47
GECo_ER_original_item28	.56	70.9	63.8	.33

Supplementary Table 13a

Item characteristics of ChatGPT-created items for GECO Emotion Management subtest.

Item number	mean score	clarity	realism	item-total correlation
GECo_EM_gpt_item1	.73	78.5	80.2	.18
GECo_EM_gpt_item2	.37	79.9	76.6	.12
GECo_EM_gpt_item3	.61	78.0	73.1	.22
GECo_EM_gpt_item4	.13	80.4	79.6	-.13
GECo_EM_gpt_item5	.15	84.3	79.5	.04
GECo_EM_gpt_item6	.33	78.5	75.4	.22
GECo_EM_gpt_item7	.82	81.1	78.3	.37
GECo_EM_gpt_item8	.77	80.7	78.0	.23
GECo_EM_gpt_item9	.55	78.4	74.5	.05
GECo_EM_gpt_item10	.33	81.8	77.6	-.05
GECo_EM_gpt_item11	.22	83.1	79.6	.04
GECo_EM_gpt_item12	.53	82.5	80.2	.24
GECo_EM_gpt_item13	.52	80.2	77.8	.16
GECo_EM_gpt_item14	.28	79.7	79.2	.03
GECo_EM_gpt_item15	.48	79.5	78.3	.18
GECo_EM_gpt_item16	.58	80.7	79.1	.16
GECo_EM_gpt_item17	.91	77.4	78.5	.23
GECo_EM_gpt_item18	.26	79.2	77.3	.05
GECo_EM_gpt_item19	.61	81.6	80.6	.17
GECo_EM_gpt_item20	.57	80.9	77.5	.02

Supplementary Table 13b

Item characteristics of original items for GECO Emotion Management subtest.

Item number	mean score	clarity	realism	item-total correlation
GECo_EM_original_item1	.77	78.0	73.6	.24
GECo_EM_original_item2	.74	78.9	73.5	.25
GECo_EM_original_item3	.58	79.0	73.9	.17
GECo_EM_original_item4	.63	78.3	75.1	.14
GECo_EM_original_item5	.73	72.8	68.7	.27
GECo_EM_original_item6	.58	76.6	78.7	.33
GECo_EM_original_item7	.51	79.1	76.0	.11
GECo_EM_original_item8	.51	80.8	78.7	.07
GECo_EM_original_item9	.37	76.5	73.4	.04
GECo_EM_original_item10	.68	75.7	75.6	.30
GECo_EM_original_item11	.49	77.5	73.6	.03
GECo_EM_original_item12	.43	80.4	78.3	.14
GECo_EM_original_item13	.58	77.1	71.0	.10
GECo_EM_original_item14	.29	78.9	76.7	.18
GECo_EM_original_item15	.47	77.6	73.0	.21
GECo_EM_original_item16	.42	76.5	71.5	.02
GECo_EM_original_item17	.45	80.4	77.3	-.06
GECo_EM_original_item18	.39	74.7	75.2	.06
GECo_EM_original_item19	.55	80.4	76.5	.28
GECo_EM_original_item20	.75	75.6	74.3	.16

Supplementary Notes 5: Similarities and differences between LLMs and human test-takers in solving ability EI items

To examine the similarities and differences between human test takers and six LLMs (ChatGPT-4, ChatGPT-o1, Copilot 365, Claude 3.5 Haiku, Gemini flash 1.5, DeepSeek V3) in solving the five EI tests, we calculated the correlations across all 105 test items between the proportions of correct responses in the human samples (i.e., the mean scores on each item) and the proportions of correct responses among the five LLMs. The datafile for this calculation can be accessed in the "supplementary data" folder in "data and analysis scripts" on OSF (file name comparison_humans_llms_osf.xlsx) : https://osf.io/mgqre/files/osfstorage .

Results indicated that, across the 105 items, the correlation between human and LLM scores was r = .46. This suggests that items with higher proportions of correct responses among humans (i.e., easier items) were also more frequently solved correctly by the LLMs. Conversely, items deemed more difficult by human standards were less frequently solved by the LLMs. The observed medium-to-large effect size suggests that humans and LLMs may rely on similar cues within the item texts to arrive at correct solutions. However, the less-than-perfect correlation also indicates notable differences between humans and LLMs, with some items being easier for humans but more challenging for LLMs, and vice versa.

For a more in-depth analysis, we calculated the differences between the proportions of correct answers provided by humans and LLMs for each item by subtracting the average human score from the average LLM score. For 15 items (14%), the difference score was negative, indicating that the average human score was higher than that of the LLM. We then examined five of these 15 items in detail for which the total LLM score was below .20 (highlighted in blue in the file mentioned above), indicating that in less than 20% of all trials across all LLMs the correct response was chosen. An inspection of the responses given by the LLMs suggests that even though they were classified as incorrect (due to the binary coding), these responses were not implausible or completely "wrong".

For example, for the GEMOK Blends item 5 describing Mark who unexpectedly sees his wife with another man, the correct response (chosen by 76% of humans) was the emotion pair "surprise and jealousy" while the LLMs consistently chose "jealousy and anger". This suggests that LLMs "missed" the description of cues related to surprise in the scenario (e.g., Mark's eyes widened, situation was unexpected) but understood the jealousy part which included some cues that could also be attributed to anger (e.g., Mark wanted to do or say something hurtful).

For the other four items, the most frequent response given by the LLMs was also frequently endorsed by test-takers (and, in some cases, was even the most commonly chosen category), although it was not the response defined as correct by the test developers. For example, in the STEM item "Reece's friend points out that her young children seem to be developing more quickly than Reece's. Reece sees that this is true. What action would be the most effective for Reece?" (item 16), the most frequently chosen option among the LLMs was option c ("Realize that children develop at different rates," expert rating 4.4 on a 6-point scale), which was also chosen by the majority of humans (63%), although the correct response was d ("Talk to a doctor about what the normal rates of development are"). In fact, the expert ratings collected by the test developers (see Supplementary Material in MacCann & Roberts, 2008) were very similar for these two responses (4.4 vs. 5.0), suggesting that both responses are plausible and effective ways of managing this scenario.

We also examined the 10 items with the highest positive difference scores (indicating that the human average was substantially lower than the LLM average score). Notably, eight of these items come from tests that utilize a theory-driven scoring approach rather than a consensus-based scoring method (i.e.,

GECo Emotion Management and Regulation subtests; GEMOK). This means that the test construction process explicitly allowed correct options to not necessarily be the most frequently selected ones (Schlegel & Mortillaro, 2019). The fact that the LLMs consistently selected the options that were theoretically defined as correct underscores their alignment with psychological theories (e.g., appraisal theory of emotion; Scherer, 2009).

Taken together, these comparisons suggest substantial agreement between LLMs and humans. However, the values for humans represent sample means, and individuals with above-average abilities may have reached even higher agreement with LLMs. In addition, there was substantial agreement among the five LLMs, with the Intraclass Correlation (ICC) across all items being .88.
